# 4-Phenyl-1,2,3-triazoles as Versatile Ligands for Cationic Cyclometalated Iridium(III) Complexes

**DOI:** 10.1021/acs.inorgchem.2c00567

**Published:** 2022-05-24

**Authors:** Alessandro Di Girolamo, Filippo Monti, Andrea Mazzanti, Elia Matteucci, Nicola Armaroli, Letizia Sambri, Andrea Baschieri

**Affiliations:** † Department of Industrial Chemistry “Toso Montanari”, University of Bologna, Bologna 40136, Italy; ‡ Istituto per la Sintesi Organica e la Fotoreattività, Consiglio Nazionale delle Ricerche (ISOF-CNR), Bologna 40129, Italy

## Abstract

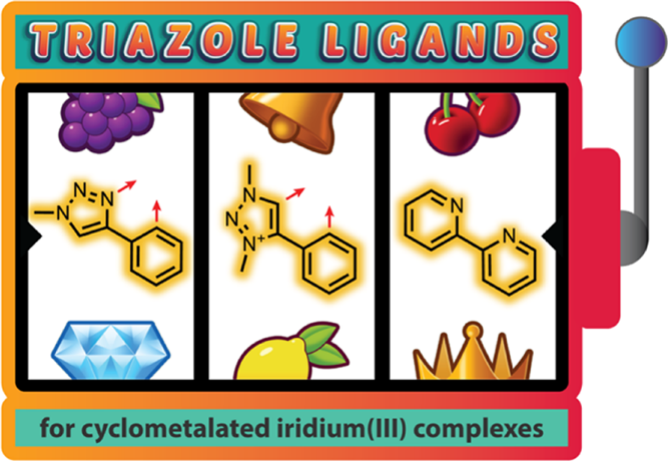

Five cationic iridium(III) complexes (**1**–**5**) were synthesized exploiting two triazole-based cyclometalating ligands, namely, 1-methyl-4-phenyl-1*H*-1,2,3-triazole (**A**) and the corresponding mesoionic carbene 1,3-dimethyl-4-phenyl-1*H*-1,2,3-triazol-5-ylidene (**B**). From the combination of these two ligands and the ancillary one, i.e., 4,4′-di-*tert*-butyl-2,2′-bipyridine (for **1**–**3**) or *tert*-butyl isocyanide (for **4** and **5**), not only the typical bis-heteroleptic complexes but also the much less explored tris-heteroleptic analogues (**2** and **5**) could be synthesized. The redox and emission properties of all of the complexes are effectively fine-tuned by the different ligands: (i) cyclometalating ligand **A** induces a stronger highest occupied molecular orbital (HOMO) stabilization compared to **B** and leads to complexes with progressively narrower HOMO–lowest unoccupied molecular orbital (LUMO) and redox gaps, and lower emission energy; (ii) complexes **1**–**3**, equipped with the bipyridine ancillary ligand, display fully reversible redox processes and emit from predominantly metal-to-ligand charge transfer (MLCT) states with high emission quantum yields, up to 60% in polymeric matrix; (iii) complexes **4** and **5**, equipped with high-field isocyanide ligands, display irreversible redox processes and high-energy emission from strongly ligand-centered triplets with long emission lifetimes but relatively low quantum yields (below 6%, both in room-temperature solution and in solid state). This work demonstrates the versatility of phenyl-triazole derivatives as cyclometalating ligands with different chelation modes (i.e., C^∧^N and C^∧^C:) for the synthesis of photoactive iridium(III) complexes with highly tunable properties.

## Abstract 2

Triazole-based cyclometalating ligands serving as both standard C^∧^N chelators (as in 4-phenyl-triazole) and C^∧^C: carbene ones (as in 4-phenyl-triazolylidene) have been used to synthesize a series of cationic iridium(III) complexes. Accordingly, unusual tris-heteroleptic complexes could be obtained by simultaneously exploiting both chelation modes. These ligands allow fine tuning of the properties of the related complexes, which exhibit luminescence all the way from blue to red and quantum yields up to 60% in a diluted polymeric matrix at 298 K.

## Introduction

Luminescent ionic transition-metal complexes, and in particular cyclometalated iridium(III) complexes, are among the most extensively explored classes of compounds for solid-state devices used for displays and lighting.^1,2^ Thanks to their exceptional versatility, they also find application as luminescent materials for biological and optical imaging,^3,4^ and in photoredox catalysis.^5,6^

Starting from the archetypal cationic complex [Ir(ppy)_2_(bpy)]^+^ (ppyH = 2-phenylpyridine; bpy = 2,2′-bipyridine) synthesized by Watts and co-workers in 1987,^7^ many derivatives have been obtained, with the aim of finding the best combinations between physical and chemical properties. Using different organic ligands (instead of the standard 2-phenylpyridine), it was possible to tune the emission color from blue to red, increase the luminescence intensity (PLQY) and the excited-state lifetime (τ), and optimize the redox potentials (*E*_ox_, *E*_red_) both in the ground state and in the excited state. These properties can be finely engineered by proper selection of the molecular structure and type of the cyclometalated and/or ancillary organic ligands.

One possible strategy is to modulate the energy of the highest occupied molecular orbital (HOMO) located on the aryl-pyridine cyclometalating ligands by adding electron-withdrawing or -donating groups on the aryl moiety. A second approach is to play with the lowest unoccupied molecular orbital (LUMO) of the complex by changing the pyridine moiety with other *N*-heterocyclic ring (e.g., *N*-heterocyclic carbene or azole).^8−11^

1,4-Disubstituted 1,2,3-triazoles can be readily synthesized using “click chemistry”. Remarkably, exploiting an alkylation–deprotonation reaction sequence, the CuAAC (Cu-catalyzed alkyne–azide cycloaddition) products can be transformed into the corresponding triazolylidenes, a peculiar type of mesoionic carbenes (MIC).^12,13^

4-Phenyl- and 4-(pyrid-2′-yl)-1,2,3-triazoles and the corresponding triazolylidenes have been widely used in recent years as monodentate or chelating ligands in coordination and organometallic chemistry. Thanks to their great versatility, metal complexes derived from such ligands (e.g., iridium(III),^14−20^ platinum(IV),^21,22^ ruthenium(II),^19,23,24^ palladium(II), gold(I),^25−27^ silver(I),^28^ and rhodium(II)^29^) have been utilized for homogeneous catalysis or as luminescent materials. When dealing with emissive heteroleptic complexes, the carbene-type chelators are normally employed as ancillary ligands.^30−32^

Recently, we successfully used 4-(pyrid-2′-yl)-1*H*-1,2,3-triazolylidene as ancillary^33^ or cyclometalating^34^ ligands to modulate the emission color of cationic cyclometalated iridium(III) complexes.

**Scheme 1:**
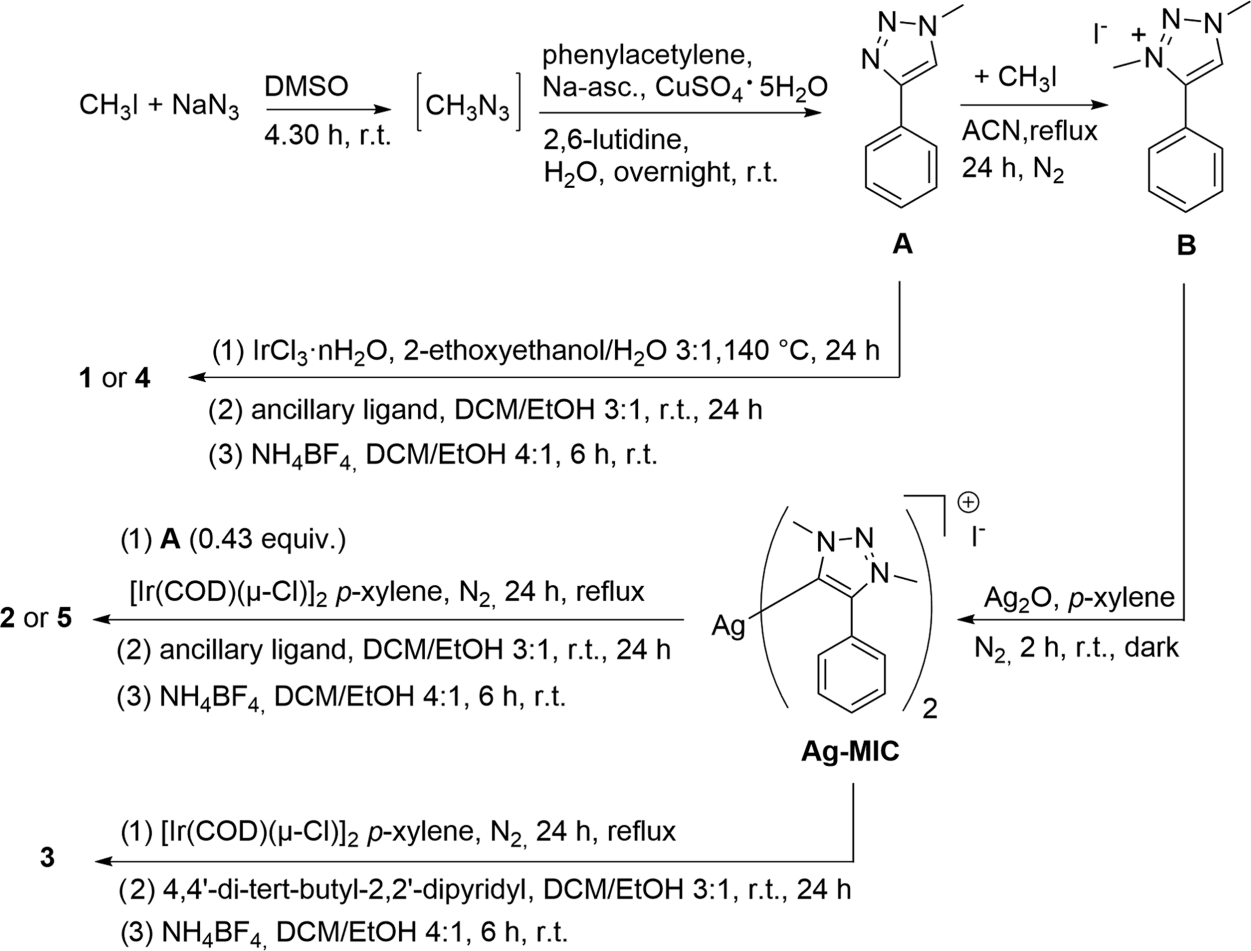
Synthesis of the Triazole **A** and the Methylated Triazolylidene **B** Used as Ligands and of the Related Complexes (**1**–**5**)

It is well known that chelating ligands containing triazoles or MICs units exhibit different σ-donor/π-acceptor properties.^35−37^ Therefore, in this work, we focused our attention on the use of 1-methyl-4-phenyl-1*H*-1,2,3-triazole (**A**) and the corresponding 1,3-dimethyl-4-phenyl-1*H*-1,2,3-triazol-5-ylidene (**B**) as versatile cyclometalating ligands for emitting cationic iridium(III) complexes (Scheme 1).

In fact, as proved later in the text, the change in the electronic nature of the bidentate ligands **A** and **B** may have significant implications on the properties of their metal complexes. Both ligands can be considered mono-anionic even if ligand **A** exploits the classical coordination as C^∧^N, while ligand **B** coordinates the metal center in C^∧^C: mode.

**Chart 1:**
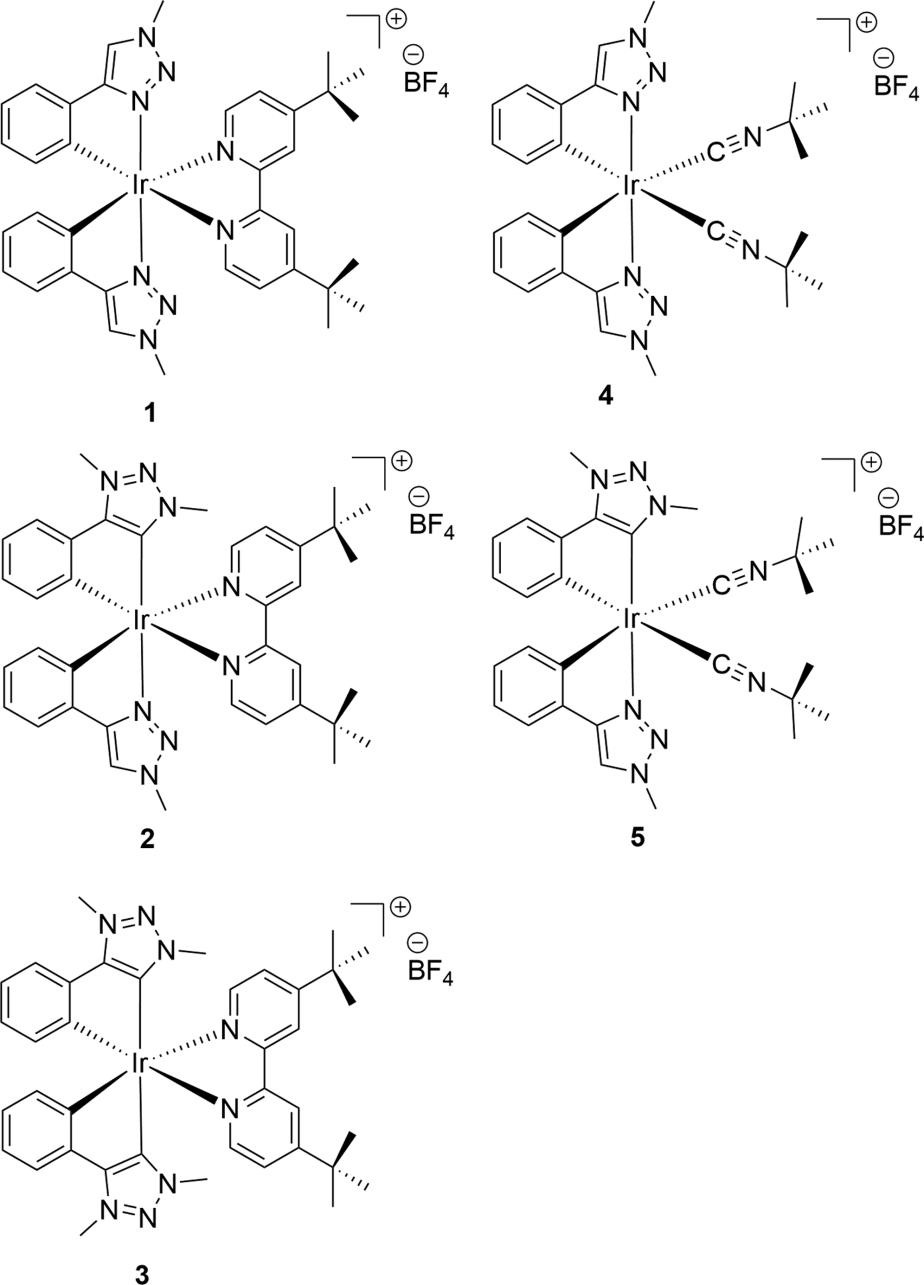
Cationic Iridium(III) Complexes Investigated in This Work

Herein, we present our results toward the synthesis and characterization of a series of new emitting iridium(III) complexes: bis-heteroleptic complexes **1**, **3**, and **4** having the classic formula [Ir(C^∧^N)_2_(N^∧^N)]^+^ and [Ir(C^∧^C:)_2_(N^∧^N)]^+^, and carrying **A** or **B** as cyclometalating ligands (Chart 1), and, for the sake of completeness, the mixed tris-heteroleptic complexes **2** and **5** having two different cyclometalating ligands, **A** and **B**, with formula [Ir(C^∧^N)(C^∧^C:)(N^∧^N)]^+^.

## Experimental Section

### General Information

Analytical-grade solvents and commercially available reagents were used as received unless otherwise stated. Chromatographic purifications were performed using 70–230 mesh silica gel. ^1^H, ^19^F, and ^13^C NMR spectra were recorded on Agilent (500 MHz for ^1^H) and Varian Mercury (400 MHz for ^1^H) spectrometers. Chemical shifts (δ) are reported in ppm relative to residual solvent signals for ^1^H and ^13^C NMR (^1^H NMR: 7.26 ppm for CDCl_3_, 5.33 ppm for CD_2_Cl_2_; ^13^C NMR: 77.0 ppm for CDCl_3_, 53.84 ppm for CD_2_Cl_2_). ^19^F NMR spectra were recorded at 470 MHz using trichlorofluoromethane as an external standard. ^13^C NMR spectra were acquired with the ^1^H broadband decoupled mode. Coupling constants are given in hertz. The abbreviations used to indicate the multiplicity of signals are: s, singlet; d, doublet; t, triplet; dd, double doublet; ddd, double double doublet; dt, double triplet; m, multiplet. The high-resolution mass spectra (HRMS) were obtained with an ESI-QTOF (Agilent Technologies, model G6520A) instrument, and the *m*/*z* values are referred to the monoisotopic mass. ESI-MS analyses were performed by direct injection of acetonitrile solutions of the compounds using a WATERS ZQ 4000 mass spectrometer.

***Caution:** Although we experienced no difficulties in handling these nitrogen-rich compounds, small-scale and best safety practices are strongly encouraged. Handle with care and pay special attention to NaN*_*3*_*as it is fatal if swallowed, in contact with skin, or if inhaled, and it may cause damage to organs (brain) through prolonged or repeated exposure, if swallowed. It is also very toxic to aquatic life with long-lasting effects*.

Ligands **A** and **B** were synthesized following a previously reported procedure with slight modifications.^33,38^

#### Synthesis of 1-Methyl-4-phenyl-1H-1,2,3-triazole (**A**)

NaN_3_ (1.43 g, 22 mmol, 1 equiv) was dissolved in DMSO (30 mL), and then MeI (1.4 mL, 22 mmol, 1 equiv) was added. The solution was stirred for 5 h at room temperature. Then, H_2_O (14 mL) was added and, to the resulting solution, phenylacetylene (2.4 mL, 22 mmol, 1 equiv), sodium ascorbate (476 mg, 2.4 mmol, 0.12 equiv), CuSO_4_·5H_2_O (70 mg, 0.28 mmol, 0.013 equiv), and 2,6-lutidine (2.6 mL, 22 mmol, 1 equiv) were added. The resulting mixture was stirred for 24 h at room temperature. After this time, H_2_O (25 mL) was added and the solution was stirred for a further 30 min. The solid was filtered and washed with H_2_O (25 mL) and NH_4_OH 30% (25 mL). Product **A** was isolated in 80% yield (2.80 g, 17.6 mmol) and used in the next step without further purifications. ^1^H NMR (CDCl_3_, 500 MHz) δ: 7.82–7.80 (m, 2H), 7.73 (s, 1H), 7.42 (t, *J* = 7.2 Hz, 2H), 7.35–7.31 (m, 1H), 4.13 (s, 3H), ^13^C NMR (CDCl_3_, 500 MHz) δ: 148.1 (C), 130.6 (C), 128.8 (CH), 128.1 (CH), 125.7 (CH), 120.5 (CH), 36.8 (CH_3_).

#### Synthesis of 1,3-Dimethyl-4-phenyl-1H-1,2,3-triazol-3-ium iodide (**B**)

**A** (1.97 g, 12.4 mmol, 1 equiv) was dissolved in acetonitrile (37 mL), and CH_3_I (4.0 mL, 64.5 mmol, 5.2 equiv) was added to the solution. The mixture was stirred for 24 h under nitrogen atmosphere. Then, the solvent was removed and the crude was purified by column chromatography on silica gel using DCM/MeOH 9:1 as an eluent, to give **B** as a white solid (3.30 g, 11.0 mmol, 88.4% yields). ^1^H NMR (CDCl_3_, 500 MHz) δ: 9.47 (s, 1H), 7.73–7.72 (m, 2H), 7.62–7.54 (m, 3H), 4.51 (s, 3H), 4.29 (s, 3H); ^13^C NMR (CDCl_3_, 500 MHz) δ: 143.0 (C), 132.0 (CH), 130.7 (CH), 129.8 (CH), 129.7 (CH), 121.7 (C), 41.3 (CH_3_), 39.10 (CH_3_).

Complexes **1** and **4** were synthesized following a previously reported procedure with slight modifications.^39−41^***Caution!** tert-Butyl isocyanide is a foul-smelling volatile liquid; therefore, ensure adequate ventilation!*

#### General Procedures for the Synthesis of Complexes **1** and **4**

Ligand **A** (55 mg, 0.34 mmol, 2.6 equiv) was dissolved in a mixture of 2-ethoxyethanol/water 3:1 (4.0 mL), and the solution was degassed with N_2_ for 20 min. Then, IrCl_3_·*x*H_2_O (40 mg, 0.13 mmol, 1 equiv) was added and the resulting mixture was refluxed for 24 h in a nitrogen atmosphere. After this time, the solvent was removed and the crude was dissolved in DCM/EtOH 3:1 (24 mL). Then, the ancillary ligand (0.18 mmol, 1.5 equiv) was added and the mixture was stirred for 24 h at room temperature. The solvent evaporated, and the crude was purified by column chromatography on silica gel with DCM/MeOH as an eluent, from 98:2 to 95:5 ratios, to give the desired products. Complexes were dissolved in DCM/EtOH 4:1 (25 mL), and NH_4_BF_4_ (450 mg, 4.3 mmol, 33 equiv) was added. The solution was stirred for 4 h at room temperature. After this time, H_2_O (10 mL) was added and the mixture was extracted with DCM (2 × 10 mL). The organic layer was dried over Na_2_SO_4_ and the solvent evaporated.

##### Complex (**1**)

Results: 76.0 mg, 0.088 mmol, yield = 68%. ^1^H NMR (500 MHz, CDCl_3_) δ: 8.19 (d, *J* = 2.2 Hz, 2H), 8.02 (d, *J* = 5.9 Hz, 2H), 7.93 (s, 2H), 7.38 (d, *J* = 9.0 Hz, 2H), 7.34 (dd, *J* = 5.9, 2.0 Hz, 2H), 6.85–6.79 (m, 2H), 6.76 (t, *J* = 8.2 Hz, 2H), 6.21 (d, *J* = 7.6 Hz, 2H), 3.96 (s, 6H), 1.42 (s, 18H); ^13^C NMR (CDCl_3_, 500 MHz) δ: 162.7 (C), 157.2 (C), 156.3 (C), 151.2 (CH), 146.2 (C), 135.6 (C), 132.4 (CH), 128.9 (CH), 128.3 (CH), 124.4 (CH), 122.5 (CH), 120.3 (CH), 119.6 (CH), 38.4 (CH_3_), 35.5 (C), 30.4 (CH_3_). HRMS (ESI-QTOF) ([M]^+^): *m*/*z* calcd for C_36_H_40_IrN_8_^+^: 775.2976; found: 775.2949.

##### Complex (**4**)

Results: 58.4 mg, 0.076 mmol, yield = 59%. ^1^H NMR (500 MHz, CDCl_3_) δ: 8.05 (s, 2H), 7.36 (d, *J* = 7.5 Hz, 2H), 6.81 (t, *J* = 8.1 Hz, 2H), 6.73 (t, *J* = 8.1 Hz, 2H), 6.04 (d, *J* = 7.5 Hz, 2H), 4.24 (s, 6H), 1.35 (s, 18H); ^13^C NMR (126 MHz, CDCl_3_) δ: 157.9 (C), 149.7 (C), 135.0 (C), 131.3 (CH), 128.2 (CH), 123.3 (CH), 122.5 (CH), 121.0 (C), 120.9 (CH), 57.9 (C), 38.6 (CH_3_), 30.3 (CH_3_); ^19^F NMR (470 MHz, CDCl_3_) δ: −152.2. HRMS (ESI-QTOF) ([M]^+^): *m*/*z* calcd for C_28_H_34_IrN_8_^+^: 673.2507; found: 673.2503.

#### General Procedures for the Synthesis of Complexes **2** and **5**

Ligand **B** (85 mg, 0.28 mmol, 4.6 equiv) and Ag_2_O (85 mg, 0.36 mmol, 6 equiv) were dispersed in *p*-xylene (5 mL), and the mixture was stirred under a nitrogen atmosphere for 2 h in the dark at room temperature. Afterward, ligand **A** (19.0 mg, 0.12 mmol, 2 equiv) and [Ir(COD)(μ-Cl)]_2_ (40.3 mg, 0.06 mmol, 1 equiv) were added and the mixture was refluxed for a further 24 h. Eventually, the resulting solid was removed by filtration on a Celite pad, and the solvent evaporated under reduced pressure. The collected solid was dissolved in DCM/EtOH 3:1 (24 mL). Then, the ancillary ligand (0.18 mmol, 1.5 equiv) was added and the mixture was stirred for 24 h at room temperature. The solvent evaporated, and the crude was purified by column chromatography on silica gel with DCM/acetone as an eluent, from 98:2 to 95:5 ratio, to afford the desired products. Complexes were dissolved in DCM/EtOH 4:1 (25 mL), and NH_4_BF_4_ (450 mg, 4.3 mmol, 70 equiv) was added. The solution was stirred for 4 h at room temperature. After this time, H_2_O (10 mL) was added and the mixture was extracted with DCM (2 × 10 mL). The organic layer was dried over Na_2_SO_4_, and the solvent evaporated.

##### Complex (**2**)

Results: 18.3 mg, 0.021 mmol, yield = 17.4%. ^1^H NMR (500 MHz, CDCl_3_) δ: 8.31 (d, *J* = 2.1 Hz, 1H), 8.29 (d, *J* = 2.1 Hz, 1H), 8.01 (d, *J* = 5.9 Hz, 1H), 7.97 (s, 1H), 7.93 (d, *J* = 5.9 Hz, 1H), 7.42 (d, *J* = 9.0 Hz, 1H), 7.37–7.29 (m, 3H), 6.94–6.85 (m, 2H), 6.80–6.73 (m, 2H), 6.44 (d, *J* = 8.7 Hz, 1H), 6.36 (d, *J* = 7.6 Hz, 1H), 4.28 (s, 3H), 4.05 (s, 3H), 3.32 (s, 3H), 1.44 (s, 9H), 1.42 (s, 9H); ^13^C NMR (126 MHz, CDCl_3_) δ: 163.2 (C), 162.4 (C), 157.1 (C), 156.7 (C), 156.2 (C), 156.1 (C), 151.3 (CH), 150.5 (CH), 150.4 (C), 148.2 (C), 146.2 (C), 135.9 (C), 135.83 (CH), 135.81 (C), 134.3 (CH), 128.7 (CH), 128.5 (CH), 124.6 (CH), 124.5 (CH), 122.9 (CH), 122.2 (CH), 122.0 (CH), 121.1 (CH), 120.5 (CH), 120.2 (CH), 120.2 (CH), 38.3 (CH_3_), 37.8 (CH_3_), 37.1 (CH_3_), 35.6 (C), 35.5 (C), 30.4 (CH_3_), 30.3_5_ (CH_3_). ESI-MS: 791 [M^+^]. HRMS (ESI-QTOF) ([M]^+^): *m*/*z* calcd for C_37_H_42_IrN_8_^+^: 789.3133; found: 789.3147.

##### Complex (**5**)

Results: 14.7 mg, 0.019 mmol, yield = 15.8%. ^1^H NMR (400 MHz, CDCl_3_) δ: 8.23 (s, 1H), 7.41 (d, *J* = 7.6 Hz, 1H), 7.30 (d, *J* = 7.0 Hz, 1H), 6.92–6.83 (m, 2H), 6.79–6.66 (m, 2H), 6.26 (d, *J* = 7.5 Hz, 1H), 6.19 (d, *J* = 6.9 Hz, 1H), 4.40 (s, 3H), 4.33 (s, 3H), 4.28 (s, 3H), 1.37 (s, 9H), 1.27 (s, 9H); ^13^C NMR (126 MHz, CDCl_3_) δ: 157.6 (C), 155.8 (C), 153.1 (C), 148.6 (C), 135.8 (C), 135.24 (C), 134.2 (CH), 133.1 (CH), 128.5 (CH), 128.3 (CH), 123.1 (CH), 123.0 (CH), 122.97 (CH), 121.0 (CH), 121.97 (CH), 57.2, 39.7 (CH_3_), 38.6 (CH_3_), 37.4 (CH_3_), 30.4 (CH_3_), 30.3 (CH_3_); ^19^F NMR (470 MHz, CDCl_3_) δ: −153.2. HRMS (ESI-QTOF) ([M]^+^): *m*/*z* calcd for C_29_H_36_IrN_8_^+^: 687.2663; found: 687.2651.

#### Synthesis of Complexes **3**

Ligand **B** (200 mg, 0.66 mmol, 2.75 equiv) and Ag_2_O (220 mg, 0.95 mmol, 4.1 equiv) were dispersed in *p*-xylene (10 mL), and the mixture was stirred under a nitrogen atmosphere for 2 h in the dark at room temperature. [Ir(COD)(μ-Cl)]_2_ (80.6 mg, 0.12 mmol, 1 equiv) was then added, and the mixture was refluxed for a further 24 h. Eventually, the resulting solid was removed by filtration on a Celite pad. The filter was washed with DCM (15 mL), and the solvent evaporated under reduced pressure. The collected solid was dissolved in DCM/EtOH 3:1 (24 mL). Then, 4,4′-di-tert-butyl-2,2′-bipyridine (80.5 mg, 0.3 mmol, 1.25 equiv) was added and the mixture was stirred for 24 h at room temperature. The solvent evaporated, and the crude was purified by column chromatography on silica gel using DCM/acetone as eluent, from 98:2 to 9:1 ratio, to give the desired products. The complex was then dissolved in DCM/EtOH 4:1 (25 mL), and NH_4_BF_4_ (1.0 g, 9.54 mmol, 40 equiv) was added. The solution was stirred for 4 h at room temperature. After this time, H_2_O (10 mL) was added and the mixture was extracted with DCM (2 × 10 mL). The organic layer was dried over Na_2_SO_4_, and the solvent evaporated.

##### Complex (**3**)

Results: 13.7 mg, 0.015 mmol, yield = 6.4%. ^1^H NMR (500 MHz, CD_2_Cl_2_) δ: 8.23 (d, *J* = 2.1 Hz, 2H), 8.08 (d, *J* = 6.0 Hz, 2H), 7.43 (d, *J* = 9.0 Hz, 2H), 7.33 (d, *J* = 7.9 Hz, 2H), 6.99 (t, *J* = 8.2 Hz, 2H), 6.78 (t, *J* = 8.2 Hz, 2H), 6.60 (d, *J* = 7.5 Hz, 2H), 4.34 (s, 6H), 3.27 (s, 6H), 1.43 (s, 18H); ^13^C NMR (126 MHz, CD_2_Cl_2_) δ: 162.3 (C), 161.5 (C), 156.7 (C), 156.2 (C), 151.1 (CH), 149.1 (C), 138.0 (CH), 136.9 (C), 128.6 (CH), 124.7 (CH), 121.7 (CH), 121.6 (CH), 119.9 (CH), 37.7 (CH_3_), 37.2 (CH_3_), 35.4 (C), 30.1 (CH_3_). HRMS (ESI-QTOF) ([M]^+^): *m*/*z* calcd for C_38_H_44_IrN_8_^+^: 803.3289; found: 803.3270.

### Electrochemical Characterization

Voltammetric experiments were performed using a Metrohm AutoLab PGSTAT 302N electrochemical workstation in combination with the NOVA 2.0 software package. All of the measurements were carried out at room temperature in acetonitrile solutions with a sample concentration of ∼0.5 mM and using 0.1 M tetrabutylammonium hexafluorophosphate (electrochemical grade, TBAPF_6_) as the supporting electrolyte. Oxygen was removed from the solutions by bubbling nitrogen. All of the experiments were carried out using a three-electrode setup (BioLogic VC-4 cell, volume range: 1–3 mL) using a glassy carbon working electrode (having an active surface disk of 1.6 mm in diameter), the Ag/AgNO_3_ redox couple (0.01 M in acetonitrile, with 0.1 M TBAClO_4_ supporting electrolyte) as the reference electrode, and a platinum wire as the counter electrode. At the end of each measurement, ferrocene was added as the internal reference. Cyclic voltammograms (CV) were recorded at a scan rate of 100 mV s^–1^. Osteryoung square-wave voltammograms (OSWV) were recorded with a scan rate of 25 mV s^–1^, an SW amplitude of ±20 mV, and a frequency of 25 Hz.

### Photophysics

The spectroscopic investigations were carried out in spectrofluorimetric-grade acetonitrile. The absorption spectra were recorded with a PerkinElmer Lambda 950 spectrophotometer. For the photoluminescence experiments, the sample solutions were placed in fluorimetric Suprasil quartz cuvettes (10.00 mm) and dissolved oxygen was removed by bubbling argon for 30 min. The uncorrected emission spectra were obtained with an Edinburgh Instruments FLS920 spectrometer equipped with a Peltier-cooled Hamamatsu R928 photomultiplier tube (PMT, spectral window: 185–850 nm). An Osram XBO xenon arc lamp (450 W) was used as the excitation light source. The corrected spectra were acquired by means of a calibration curve, obtained using an Ocean Optics deuterium–halogen calibrated lamp (DH-3plus-CAL-EXT). The photoluminescence quantum yields (PLQYs) in solution were obtained from the corrected spectra on a wavelength scale (nm) and measured according to the approach described by Demas and Crosby,^42^ using an air-equilibrated water solution of tris(2,2′-bipyridyl)ruthenium(II) dichloride as reference (PLQY = 0.028).^43^ The emission lifetimes (τ) were measured through the time-correlated single photon counting (TCSPC) technique using a HORIBA Jobin Yvon IBH FluoroHub controlling a spectrometer equipped with a pulsed NanoLED (λ_exc_ = 280 and 370 nm) or SpectraLED (λ_exc_ = 370 nm) as the excitation source and a red-sensitive Hamamatsu R-3237-01 PMT (185–850 nm) as the detector. The analysis of the luminescence decay profiles was accomplished with the DAS6 Decay Analysis Software provided by the manufacturer, and the quality of the fit was assessed with the χ^2^ value close to unity and with the residuals regularly distributed along the time axis. For the determination of emission lifetimes longer than 100 μs, the μF920H pulsed lamp by Edinburgh Instruments was coupled with the above-mentioned FLS920 spectrometer; data analysis was performed with the software provided by the manufacturer. To record the 77 K luminescence spectra, samples were put in quartz tubes (2 mm inner diameter) and inserted into a special quartz Dewar flask filled with liquid nitrogen. Poly(methyl methacrylate) (PMMA) films containing 1% (w/w) of the complex were obtained by drop-casting, and the thickness of the films was not controlled. Solid-state PLQY values were calculated by corrected emission spectra obtained from an Edinburgh FLS920 spectrometer equipped with a barium sulfate-coated integrating sphere (diameter of 4 in.) following the procedure described by Würth et al.^44^ Experimental uncertainties are estimated to be ±8% for τ determinations, ±10% for PLQYs, and ±2 and ±5 nm for absorption and emission peaks, respectively.

### Computational Details

Density functional theory (DFT) calculations were carried out using the B.01 revision of the Gaussian 16 program package^45^ in combination with the M06 global-hybrid meta-GGA exchange-correlation functional.^46,47^ The fully relativistic Stuttgart/Cologne energy-consistent pseudopotential with multielectron fit was used to replace the first 60 inner-core electrons of the iridium metal center (i.e., ECP60MDF) and was combined with the associated triple-ζ basis set (i.e., cc-pVTZ-PP basis).^48^ On the other hand, the Pople 6-31G(d,p) basis was adopted for all other atoms.^49,50^ All of the reported complexes were fully optimized without symmetry constraints, using a time-independent DFT approach, in their ground state (S_0_) and lowest triplet states; all of the optimization procedures were performed using the polarizable continuum model (PCM) to simulate acetonitrile solvation effects.^51−53^ Frequency calculations were always used to confirm that every stationary point found by geometry optimizations was actually a minimum on the corresponding potential-energy surface (no imaginary frequencies). To investigate the nature of the emitting states, geometry optimizations and frequency calculations were performed at the spin-unrestricted UM06 level of theory (imposing a spin multiplicity of 3), using the S_0_ minimum-energy geometry as an initial guess. The emission energy from the lowest triplet excited states was estimated by subtracting the SCF energy of the emitting state (T_n_) in its minimum conformation from that of the singlet ground state having the same geometry and equilibrium solvation of T_n_. Time-dependent DFT calculations (TD-DFT),^54,55^ carried out at the same level of theory used for geometry optimizations, were used to calculate the first 16 triplet excitations, and their nature was assessed with the support of natural transition orbital (NTO) analysis.^56^ Charge decomposition analysis and orbital-interaction diagram were performed using Multiwfn 3.8—A Multifunctional Wavefunction Analyzer.^57^ All of the pictures showing molecular geometries, orbitals, and spin-density surfaces were created using GaussView 6.^58^

## Results and Discussion

### Synthesis

According to previously reported procedures,^38^ 1-methyl-4-phenyl-1*H*-1,2,3-triazole **A** was synthesized in an efficient one-pot two-step reaction, using a copper-mediated azide–alkyne cycloaddition (CuAAC) of commercially available phenylacetylene with methyl azide, prepared *in situ* from methyl iodide and NaN_3_. After 24 h, the white solid product was recovered by simple filtration in essentially pure form. To get the triazolium salt, 1,3-dimethyl-4-phenyl-1*H*-1,2,3-triazol-3-ium iodide (**B**), precursor of the triazolylidene, methyl iodide, was used to methylate **A**. The methylation occurs at N-3 of the triazole ring giving **B** in 88.4% yield (Scheme 1). The structure of the latter was confirmed by X-ray analysis and one-dimensional (1D) NMR experiments. The N–N bond distances were found to be almost identical (i.e., 1.32 and 1.31 Å), as well as the N–CH_3_ ones (i.e.,1.46 and 1.46 Å; see detailed crystal data in the Supporting Information). These results are in agreement with a previously reported structure.^59^

The obtained ligands were then exploited in different cyclometallation reactions to get the desired iridium(III) complexes. In detail, complexes **1** and **4** were obtained by means of the most straightforward route that involves the direct cyclometalation of iridium(III) chloride hydrate (IrCl_3_·*x*H_2_O) with ligand **A**, as previously reported.^39^ The chloro-bridged dimer thus obtained was reacted with the two different ancillary ligands to get, after anion exchange with NH_4_BF_4_, the iridium complexes **1** and **4** in 68 and 59% overall yields, respectively (Scheme 1).

On the other hand, we set up a new one-pot procedure using the triazolium salt **B** as a cyclometalating agent to obtain the corresponding cationic complexes of iridium(III). First of all, we optimized the synthesis of the carbene by reacting ligand **B** with Ag_2_O in *p*-xylene to give the corresponding silver(I) triazolylidene complex (**Ag-MIC**) (Scheme 1). The success of this step was checked by isolating the carbene in a test reaction: ^1^H NMR analysis confirmed the complete deprotonation of the C–H of the triazole ring (Figure S7). Then, [Ir(COD)(μ-Cl)]_2_, an Ir-source more reactive than IrCl_3_, was added *in situ* to the obtained carbene. By means of a silver–iridium transmetalation step and after the addition of the ancillary ligand, 4,4′-di-*tert*-butyl-2,2′-bipyridine, and treatment with an excess of NH_4_BF_4_, we obtained the mononuclear complex **3** as tetrafluoroborate salt in 6.4% overall yields. We also tried to obtain the corresponding complex having *tert*-butyl isocyanide as an ancillary ligand, but the very low reaction yield did not allow a suitable product purification for the subsequent photophysical characterization.

The procedure to get tris-heteroleptic complexes **2** and **5** was more elaborated. A careful screening was necessary to find the optimal ratio among the ligands to maximize the tris-heteroleptic complexes yields. Ligand **A** was added to the **Ag-MIC** solution in such an amount to have a molar ratio **A**/**B** = 3/7 (0.43 equiv **A** to **B**), followed by the addition of [Ir(COD)(μ-Cl)]_2_, the proper ancillary ligand, and NH_4_BF_4_, as described for complex **3**. After chromatographic purification, complexes **2** and **5** were isolated in 17.4 and 15.8% overall yields, respectively (Scheme 1)

All compounds were fully characterized by NMR spectroscopy (Figures S1–S25) and mass spectrometry.

### Theoretical Calculation: Ground-State Properties

For a proper understanding of the electronic structure and optical properties of **1**–**5**, DFT and TD-DFT calculations were carried out using the M06 hybrid meta-GGA exchange-correlation functional.^46,47^ All complexes were fully optimized in their ground state taking into account acetonitrile solvation effects, using the polarizable continuum model (PCM).^51−53^ The efficacy of the adopted computational approach has already been validated on similar systems, as proved by several publications in the field.^60,61^

**Figure 1:**
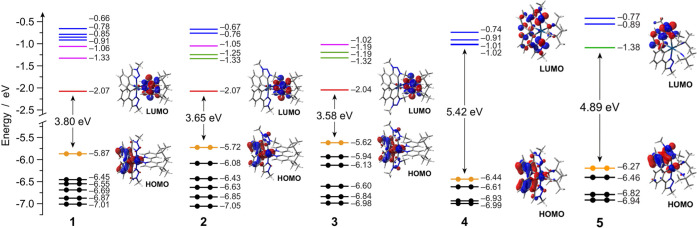
Energy diagram showing the energy values of the frontier Kohn–Sham molecular orbitals of **1**–**5** in acetonitrile. For some relevant orbitals, the corresponding isosurface is also displayed for the sake of clarity (isovalue = 0.04 e^1/2^ bohr^–3/2^). Along the series, relevant orbitals with similar topology are plotted with the same color for easier comparison.

The energy diagrams and the frontier molecular orbitals of **1**–**5** are reported in Figure 1. As commonly observed in other cationic iridium(III) complexes, also for all of the complexes of the present series, the HOMO is mainly localized on the iridium d orbitals and on the phenyl moieties of the cyclometalating ligands.^1,60^ Notably, the cyclometalated phenyl-triazole is able to induce a larger ligand-field splitting when coordinated as a standard C^∧^N cyclometalated ligand, as suggested by a more pronounced HOMO stabilization. Indeed, for complexes **1**–**3** (having the same bpy-type ancillary ligand), such stabilization is maximized in **1**, equipped with two phenyl-triazole C^∧^N cyclometalating ligands, and becomes weaker and weaker as long as such ligands are gradually replaced by phenyl-triazolylidene C^∧^C: analogues as in **2** and **3**, respectively (Figure 1).

Actually, carbene-based ligands are known to be strong σ donors,^62−66^ and the lower ligand-field splitting exerted by the phenyl-triazolylidene C^∧^C: ligand with respect to the phenyl-triazole C^∧^N equivalent may appear peculiar. To better clarify this finding, an orbital-interaction diagram is reported in Figure S26, showing the role of the two C^∧^N and C^∧^C: cyclometalating ligands in determining the electronic properties of complexes **1** and **3**, respectively.^67^ The smaller ligand-field splitting observed for complex **3** is mainly due to the little higher π-donor nature of the phenyl-triazolylidene ligand,^68^ which better destabilizes the fully occupied pseudo-t_2g_* orbitals to which the HOMO belongs (Figure S26). A similar effect is also observed in **4** and **5**, but their HOMO is already heavily stabilized by the presence of the extremely strong-field *tert*-butyl isocyanide ligands, as already well reported in the literature.^69−71^

On the contrary, the LUMO has a different nature along the series. In fact, for complexes **1**–**3** (which are all equipped with the dtbbpy ancillary ligand), the LUMO is fully localized on the lowest-lying π* orbital of such N^∧^N ligand and its energy is virtually unaffected along the series; as a consequence, the HOMO–LUMO energy gap of these complexes is substantially determined by HOMO stabilization (i.e., **1** > **2** > **3**). Conversely, in the case of **4** and **5** (lacking low-lying π* orbitals on the ancillary ligands), the LUMO is centered on the cyclometalating ligands. As depicted in Figure 1 for **5**, the lowest-energy π* orbital of the phenyl-triazolylidene C^∧^C: ligand is lower in energy by ∼0.5 eV with respect to the one centered on the phenyl-triazole C^∧^N counterpart, so the former accommodates the LUMO and the latter the LUMO+1. Since complex **4** only has phenyl-triazole C^∧^N cyclometalating ligands, its LUMO is very high in energy, resulting in an even wider HOMO–LUMO gap (Figure 1). Such virtual orbitals are also present in **1**–**3**, but they can be found at a much higher energy with respect to the dtbbpy-centered LUMO; therefore, they are expected not to play an important role in the electrochemistry and photophysics of these complexes (see below).

### Electrochemistry

**Figure 2:**
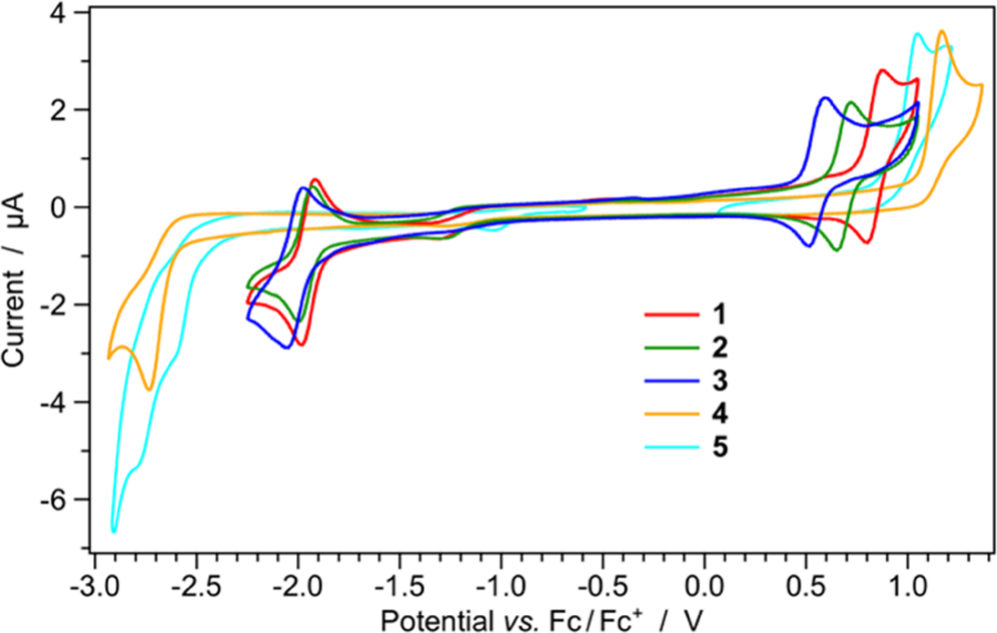
Cyclic voltammograms of complexes **1**–**5** (0.5 mM) in acetonitrile solution at 298 K.

To explore how the different chelation mode of the phenyl-triazole ligands affects the electronic properties of the related cyclometalated iridium(III) complexes, cyclic and square-wave voltammetry experiments were carried out in room-temperature acetonitrile solutions (Figures 2 and S27, respectively) and the recorded redox potentials are reported in Tables 1 and S2, relative to the Fc/Fc^+^ couple (see the Experimental Section for further details).

**Table 1:** Electrochemical Data of **1**–**5** in Acetonitrile Solution (0.5 mM) + 0.1 M TBAPF_6_ at 298 K

	*E*_ox_ (Δ*E*_p_)^a^	*E*_red_ (Δ*E*_p_)^a^	Δ*E*_redox_^b^
	[*V* (mV)]	[*V* (mV)]	[V]
**1**	+0.837 (72)	–1.948 (65)	2.785
**2**	+0.687 (68)	–1.964 (64)	2.651
**3**	+0.553 (78)	–2.014 (73)	2.567
**4**	+1.16 (irr.)	–2.70 (irr.)	3.86
**5**	+1.01 (irr.)	–2.57 (irr.), −2.76 (irr.)	3.58

a The reported potential values are obtained by cyclic voltammetry and reported vs the ferrocene/ferrocenium couple, used as the internal reference. The value in parentheses is the peak-to-peak separation (Δ*E*_p_); redox processes are reversible, unless otherwise stated (irr.).

b Δ*E*_redox_ = *E*_ox_ – *E*_red_.

As shown in Figure 2, all redox processes involving the three complexes equipped with the 2,2′-bipyridine ancillary ligand (i.e., **1–3**) are fully reversible, while irreversible processes are observed when ancillary isocyanide ligands are used (i.e., **4** and **5**), as commonly found in the literature.^61,69,72^ For all complexes, the oxidation process can be formally attributed to the Ir(III)/Ir(IV) redox couple, as well known from the literature^1,60^ and already confirmed by DFT calculations (see the previous section). As a consequence, along the series, the oxidation potentials strongly vary depending on the ligand-field strength of the different cyclometalating ligands and ancillary ones. Indeed, among complexes **1**–**3** equipped with the same dtbbpy ancillary ligand, when the phenyl-triazole chelates the iridium center in the standard C^∧^N cyclometalation mode, the ligand-field strength is maximized and the oxidation potential is the highest along the series (i.e., +0.837 V in complex **1**). On the contrary, when the same ligand is methylated and coordinates the metal ion as a mesoionic carbene (i.e., using the C^∧^C: cyclometalation mode), the HOMO stabilization is less pronounced and a lower oxidation potential is observed (i.e., +0.553 V, as in **3**). The oxidation potential is intermediate in the case of complex **2**, in which one phenyl-triazole and one phenyl-triazolylidene are used as C^∧^N and C^∧^C: cyclometalating ligands (i.e., *E*_ox_ = +0.687 V, see Table 1). Such results are in excellent agreement with DFT calculation since a HOMO stabilization of 0.10 and 0.15 eV is theoretically predicted when passing from **3** to **2** and from **2** to **1**, in line with an anodic shift in oxidation potentials of 0.13 and 0.15 V, respectively.

An analogous scenario is observed for **4** and **5**, equipped with stronger-field *tert*-butyl isocyanide ancillary ligands. In fact, despite much higher oxidation potentials, the same anodic shift of ∼0.15 V is observed when replacing one phenyl-triazolylidene cyclometalating ligand with a stronger phenyl-triazole analogue, as occurs when passing from **5** to **4**.

As far as the cathodic region is concerned, all complexes equipped with the dtbbpy ancillary ligand (i.e., **1**–**3**) display virtually identical reduction potentials (*E*_red_ = (−1.97 ± 0.03) V, see Table 1). This is because, in these three complexes, the reduction process is centered on such N^∧^N ancillary ligand, as indicated by DFT calculations (Figure 1). On the contrary, in the case of **4** and **5**, reduction occurs at much more negative potentials due to the lack of low-lying π* orbitals on the ancillary ligands; consequently, the reduction processes involve the cyclometalating ligands. Since the C^∧^C: phenyl-triazolylidene ligand displays lower-lying π* orbitals with respect to the C^∧^N phenyl-triazole analogue (see Figure 1), the reduction of **5** is recorded at −2.57 V, while a further cathodic shift of 0.13 V is observed in the reduction potential of **4**. Notably, in the case of **5**, a second reduction process is also detected at −2.76 V and it can be reasonably attributed to the reduction of the phenyl-triazole ligand, after the first reduction of the phenyl-triazolylidene moiety.

### Photophysical Properties and Excited-State Calculations

**Figure 3:**
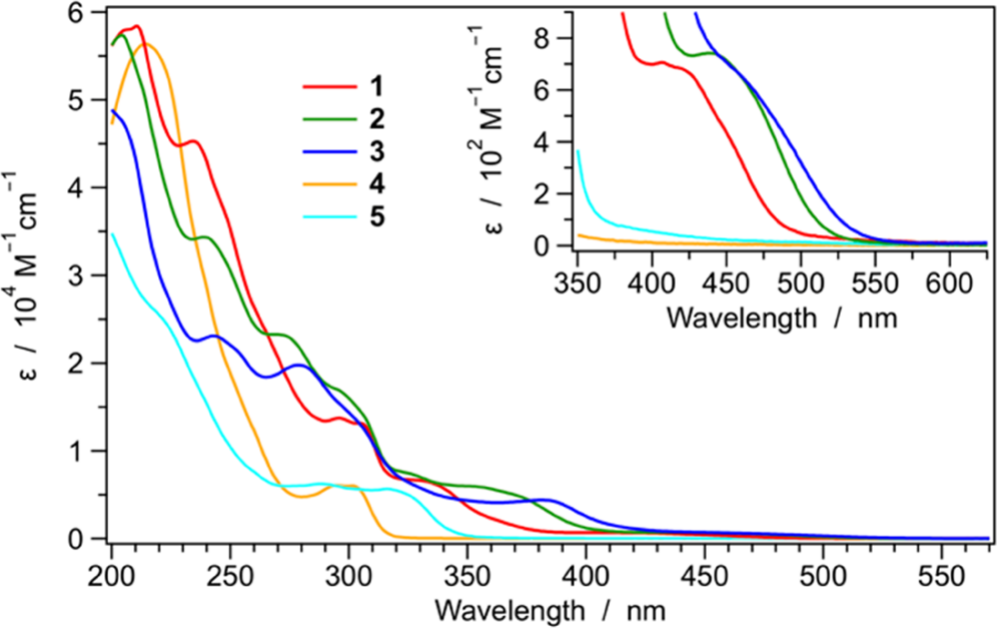
Absorption spectra of complexes **1–5** in room-temperature acetonitrile solution. Lowest-energy transitions are magnified in the inset.

The UV–vis absorption spectra of complexes **1**–**5** were recorded in acetonitrile solution at room temperature (Figure 3) and compared with their counterparts recorded in less polar dichloromethane solution (Figure S28).

As usually observed for other cyclometalated iridium(III) complexes, the main absorption bands in the region between 200 and 300 nm can be attributed to spin-allowed ligand-centered (LC) π–π* transitions located on both the cyclometalating and the ancillary ligands; at longer wavelengths (300–400 nm), the weaker and broader bands can be assigned to charge transfer transitions with mixed ligand-to-ligand, intraligand, or metal-to-ligand charge transfer (LLCT/ILCT/MLCT) nature.^1,60^

It should be emphasized that all of the complexes equipped with the dtbbpy ancillary ligand (i.e., **1**–**3**) show distinct absorption bands at λ > 330 nm with ε ≈ 4–6 × 10^3^ M^–1^ cm^–1^, mainly due to the spin-allowed HOMO–LUMO transition (see above). The energy of such ^1^MLCT absorption bands follows the order **1** > **2** > **3**, according to both DFT and electrochemical findings (Figure 1 and Table 1, respectively). On the other hand, this type of transition is totally absent in complexes **4** and **5**, equipped with isocyanide ancillary ligands lacking low-energy π* orbitals, and their absorption profiles drop to zero at λ > 320 and 350 nm, respectively (Figure 3). A very similar scenario is also observed in less polar dichloromethane solution, especially at λ < 350 nm, where ligand-centered transitions are expected (Figure S28).

In the inset of Figure 3, the lowest-energy absorption bands of the complexes are magnified. They are assigned to the direct population of the lowest triplet state by the formally spin-forbidden S_0_ → T_1_ transition. Despite such bands becoming partially allowed due to the high spin–orbit coupling of the iridium center,^1^ they remain extremely weak (ε < 800 M^–1^ cm^–1^) and are only detectable for complexes **1**–**3**, showing a T_1_ with a strongly pronounced ^3^MLCT character (see below). Notably, such S_0_ → T_1_ bands are slightly red-shifted in dichloromethane solution, demonstrating the influence of solvent polarity on such ^3^MLCT transitions (Figure S28).

**Figure 4:**
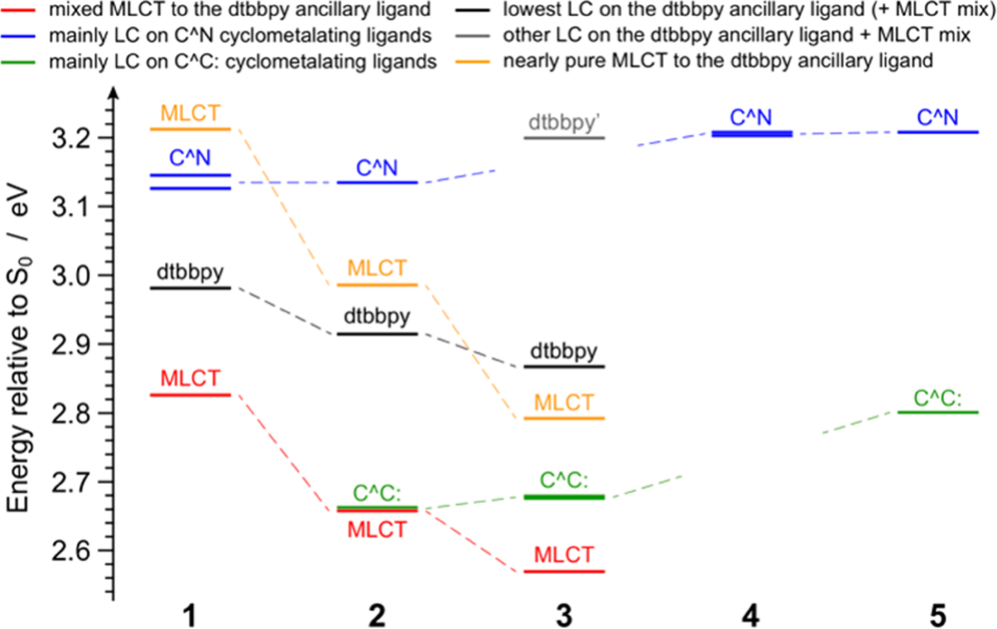
Energy diagram of the lowest-lying triplet states for complexes **1**–**5**, computed in acetonitrile as vertical excitations from the respective ground-state minimum-energy geometries.

To get a deeper insight into the excited-state properties of these complexes, the lowest-lying triplet states of **1**–**5** were investigated by means of TD-DFT methods. Tables S2–S6 summarize the lowest triplet excitations of **1**–**5**, depicted as couples of natural transition orbitals (NTOs).^56^ For the sake of clarity, Figure 4 reports a compact representation of the triplet-state energy landscape at the Franck–Condon region for all of the investigated complexes.

TD-DFT calculations further confirm that the lowest-energy absorption band recorded in the 400–500 nm region for complexes **1**–**3** (Figure 3, inset) corresponds to the S_0_ → T_1_ transition, having a predominant MLCT character (Tables S2–S4). As shown in Figure 4, calculations nicely predict that the energy of this transition gradually decreases along the series (i.e., 2.83 > 2.66 > 2.57 eV for **1**, **2**, and **3**, respectively). Moreover, such theoretical values correlate well with the experimental absorption maxima (i.e., 3.04 > 2.82 > 2.68 eV) with a minor underestimation of ∼0.15 eV; therefore, complexes **1**–**3** are expected to emit from such MLCT state. It should be emphasized that, for **2**, T_1_ is nearly isoenergetic with T_2_ (Δ*E* ≈ 5 meV), which is a ligand-centered state located on the phenyl-triazolylidene ligand. In **3**, due to the presence of two of such C^∧^C: chelators, both T_2_ and T_3_ are localized on such ligands. However, both these upper-lying states are not expected to play a relevant role in the photophysics of **3** because the MLCT state is strongly stabilized.

A completely different excited-state scenario is observed for **4** and **5**. Indeed, due to the absence of the dtbbpy ancillary ligand offering a low-lying π* orbital, the lowest-energy transitions are only those localized on the cyclometalating ligands. These are found at higher energy with respect to the corresponding ones in **1**–**3**, due to the stronger ligand field of the *tert*-butyl isocyanides. In **4**, T_1_ is located 3.20 eV above S_0_ and it is nearly degenerate with T_2_ since they are both centered on the equivalent phenyl-triazole C^∧^N ligands. On the other hand, the tris-heteroleptic complex **5** displays a lower-lying triplet located on its phenyl-triazolylidene C^∧^C: ligand (i.e., T_1_) and an upper-lying one centered on the remaining phenyl-triazole C^∧^N ligand (i.e., T_2_, Δ*E* = 0.41 eV, see Figure 4). Consequently, both **4** and **5** are expected to show a blue-shifted emission compared to **1**–**3**, with **4** exhibiting the most shifted band.

**Figure 5:**
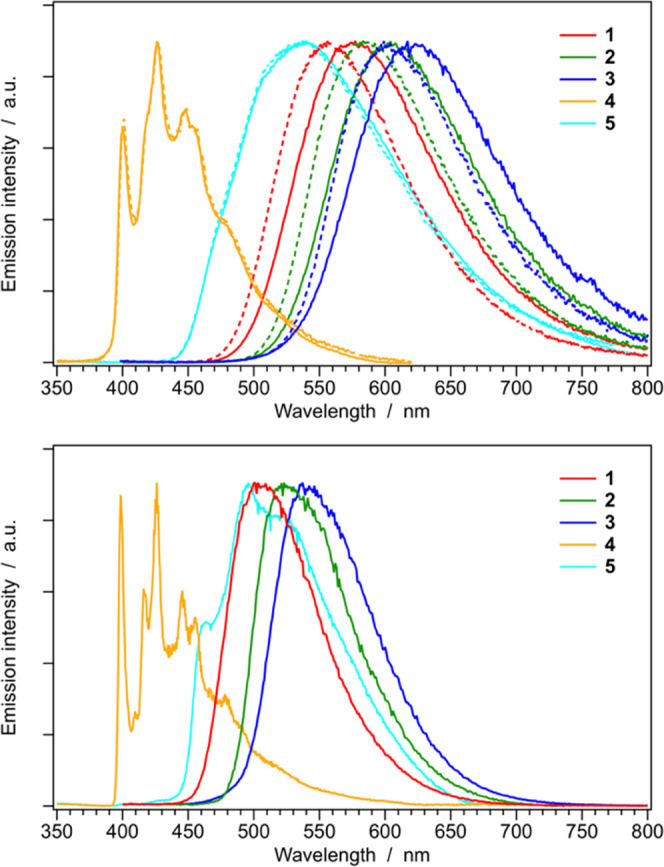
Normalized emission spectra of complexes **1–5** in acetonitrile (solid) and in dichloromethane (dashed) solutions at 298 K (top) and in butyronitrile glass at 77 K (bottom). Sample concentration: ≈20 μM.

Normalized emission spectra of **1**–**5** in room-temperature acetonitrile and dichloromethane solutions are shown in Figure 5 (top), while the same spectra recorded in butyronitrile glass at 77 K are reported in Figure 5 (bottom), to allow a direct comparison. The corresponding luminescence properties and photophysical parameters are listed in Table 2.

**Table 2:** Luminescence Properties and Photophysical Parameters of Complexes **1**–**5** in Diluted Solutions

	CH_3_CN oxygen-free solution, 298 K					CH_2_Cl_2_ oxygen-free solution, 298 K					BuCN rigid matrix, 77 K	
	*λ*_em_^a^	PLQY^a^	τ^b^	*k*_r_^c^	*k*_nr_^d^	λ_em_^e^	PLQY^e^	τ^b^	*k*_r_^c^	*k*_nr_^d^	*λ*_em_^a^	τ^b^
	[nm]	[%]	[μs]	[10^4^ s^–1^]	[10^5^ s^–1^]	[nm]	[%]	[μs]	[10^4^ s^–1^]	[10^5^ s^–1^]	[nm]	[μs]
**1**	575	27.0	0.771	35.1	9.46	556	41.4	0.987	41.9	5.94	505	4.06
**2**	605	14.8	0.481	30.7	17.7	583	30.6	0.834	36.7	8.32	523	8.63
**3**	623	8.9	0.358	24.9	25.4	604	20.6	0.681	30.2	11.7	542	13.1
**4**	400, 427, 447	0.9	3.71	0.247	2.67	401, 426, 449	0.5	1.55	0.347	6.43	399, 426, 446	157
**5**	538	5.6	39.6	0.140	0.238	539	5.7	46.5	0.122	0.203	464^sh^, 495, 520^sh^	286

a *λ*_exc_ = 280 nm for **4** and **5**, 340 nm for **1**–**3**.

b *λ*_exc_ = 280 nm for **4** and **5**, 370 nm for **1**–**3**.

c Radiative constant: *k*_r_ = PLQY/τ.

d Nonradiative constant: *k*_nr_ = 1/τ – *k*_r_.

e *λ*_exc_ = 280 nm.

According to TD-DFT calculations, complexes **1**–**3** emit from ^3^MLCT states, as also experimentally proven by (i) the broad and unstructured emission bands observed both at room temperature and at 77 K; (ii) the remarkably red-shifted emission observed in more polar acetonitrile solution, compared to dichloromethane; (iii) the considerable rigidochromic shift observed upon cooling (compare Figure 5 top and bottom); and (iv) relatively high radiative rate constants (i.e., *k*_r_ = (3.0 ± 0.5) × 10^5^ s^–1^, see Table 2).

Moreover, as suggested by comparable values of *k*_r_, all of these complexes emit from the same type of ^3^MLCT state, as also confirmed by unrestricted DFT calculations carried out to optimize the lowest triplet state of such molecules (Figure S29). Consequently, the gradual decrease in the photoluminescence quantum yields observed when passing from **1** to **3** (i.e., PLQY = 0.270, 0.148, and 0.089 for **1**, **2**, and **3** in acetonitrile, respectively, Table 2) is basically due to an increase in nonradiative rate constants, due to the energy-gap law. Indeed, in dichloromethane solution (where blue-shifted emissions are observed), a remarkable reduction in the *k*_nr_ values of **1**–**3** is observed, if compared to the corresponding nonradiative rate constants found in acetonitrile solutions, leading to considerably higher PLQYs (Table 2). In addition, as expected from absorption data and TD-DFT predictions, the energy of the ^3^MLCT emission bands follows the order **1** > **2** > **3**. Such a trend is nicely corroborated by unrestricted DFT calculations, which estimate emission energies of 1.99, 1.89, and 1.71 eV for **1**, **2**, and **3** with acetonitrile implicit solvation, respectively, to be compared with experimental mean-photon energies of 2.08, 1.98, and 1.92 eV (Figure S30).

Since TD-DFT calculations indicate that **2** displays virtually isoenergetic T_1_ and T_2_ at the Franck–Condon region (see before and Figure 4), we decided to fully optimize also the latter triplet to properly understand the role of T_2_ in the excited-state deactivation of **2**. Figure S31 reports an energy diagram showing that, upon relaxation, the adiabatic energy gap between T_1_ and T_2_ increases to 79 meV, leading to a population of the lowest triplet (the ^3^MLCT – T_1_) of more than 95% at 298 K. Therefore, the role of T_2_ (the ^3^LC state centered on the C^∧^C: phenyl-triazolylidene ligand) can be safely assumed not to be responsible for emission. Nevertheless, it is extremely likely that T_2_ may be also populated after excitation and that it undergoes ultrafast internal conversion to T_1_ within a ps time scale, as already demonstrated by several transient-absorption experiments carried out on similar systems.^73−77^

On the other hand, the emission of **4** is due to a strongly ^3^LC state located on the phenyl-triazole cyclometalating ligands, as experimentally corroborated by: (i) strongly vibrationally resolved emission profiles both at room temperature and 77 K; (ii) the lack of any solvatochromism, if comparing the emission spectra in acetonitrile and dichloromethane solutions; (iii) the absence of any shift on passing from 298 to 77 K solution; and (iv) long excited-state lifetime in the μs time domain, with a low radiative rate constant (Figure 5 and Table 2). Such a scenario is substantially confirmed by unrestricted DFT calculations (Figure S29), which only slightly underestimate the emission of **4** to occur at 2.69 eV in acetonitrile solution—to be compared with an experimental mean-photon energy of 2.79 eV (Figure S30).

In the case of complex **5**, the scenario seems to be more controversial since a broad emission band is observed at room temperature, suggesting a ^3^MLCT emission. Anyway, the emission spectrum of **5** is virtually solvent insensitive (Figure 5, top) and *k*_r_ is around 200 times lower with respect to that of **1**–**3** (Table 2). These latter evidences, together with a more structured emission profile at 77 K (Figure 5), strongly indicate a ^3^LC emitting state, as predicted by TD-DFT calculations (Figure 4). Indeed, unrestricted DFT calculations unquestionably demonstrate that the emitting triplet (i.e., T_1_) is a ^3^LC state centered on the C^∧^C: phenyl-triazolylidene ligand (having the same nature as T_2_ in complex **2**). The reason for the broad and poorly structured emission band observed for complex **5** in room-temperature solution is due to the remarkable excited-state distortions occurring in the C^∧^C: ligand upon excited-state relaxation (see Figure S32 for further details).

**Figure 6:**
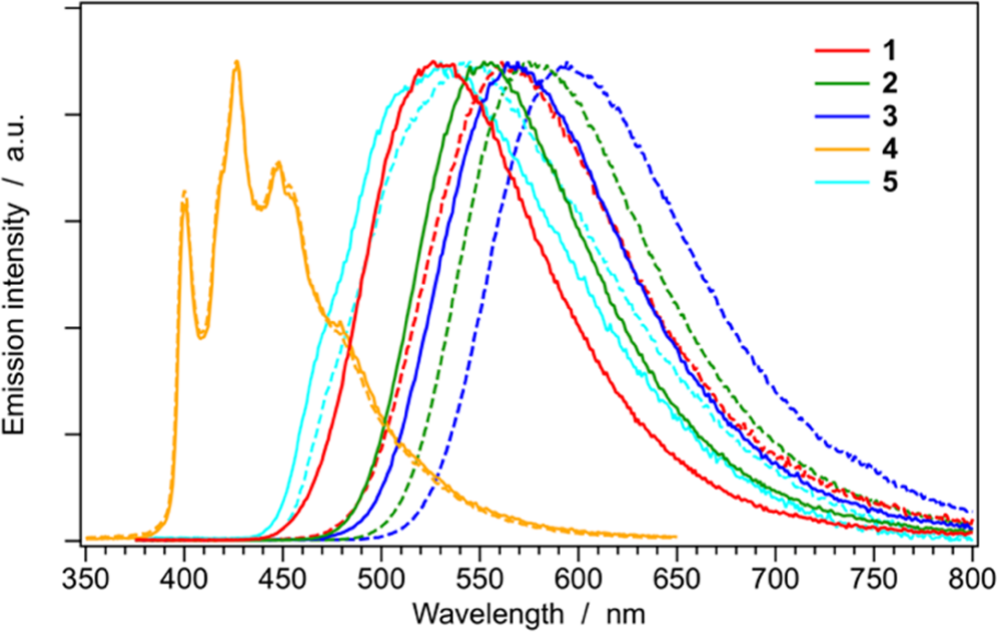
Normalized emission spectra of complexes **1–5** in 1% w/w PMMA matrix (full) and in neat film (dashed) at 298 K.

The photophysical characterization of the complexes was also carried out in solid state by (i) dispersing the emitters in a poly(methyl methacrylate) (PMMA) matrix at a concentration of 1% by weight and (ii) as neat films. The emission spectra in solid state were recorded at 298 K (open to air) and are reported in Figure 6; the associated photophysical parameters are summarized in Table 3.

**Table 3:** Luminescence Properties and Photophysical Parameters of Complexes **1**–**5** in Solid State at 298 K

	1% PMMA matrix					neat film				
	λ_em_^a^	PLQY^b^	τ^c^	*k*_r_^d^	*k*_nr_^e^	λ_em_^a^	PLQY^b^	τ^c^	*k*_r_^d^	*k*_nr_^e^
	[nm]	[%]	[μs]	[10^4^ s^–1^]	[10^5^ s^–1^]	[nm]	[%]	[μs]	[10^4^ s^–1^]	[10^5^ s^–1^]
**1**	530	59.6	1.06	56.2	3.81	562	7.5	0.496	15.1	18.6
**2**	554	59.7	1.32	45.2	3.05	577	18.1	0.503	36.0	16.3
**3**	566	48.4	1.19	40.6	4.34	595	11.3	0.636	17.8	13.9
**4**	400, 427, 448	2.7	14.6	0.186	0.666	401, 428, 448	0.6	2.23	0.269	4.46
**5**	510^sh^, 533	6.0	49.4	0.121	0.190	542	4.2	32.5	0.129	0.295

a *λ*_exc_ = 300 nm (and 370 nm for **1**–**3** only).

b Photoluminescence quantum yield determined by integrating sphere.

c *λ*_exc_ = 280 nm for **4** and **5**, 370 nm for **1**–**3**.

d Radiative constant: *k*_r_ = PLQY/*τ*.

e Nonradiative constant: *k*_nr_ = 1/τ – *k*_r_.

In diluted PMMA matrix, all of the complexes equipped with the dtbbpy ancillary ligand (i.e., **1**–**3**) exhibit a remarkable increase in their photoluminescence quantum yields, if compared to room-temperature acetonitrile solution (e.g., PLQY ≈ 60% for **1** and **2**, Table 2); moreover, the energy of their ^3^MLCT emission bands is somehow in between the one recorded in solution at 298 K and the one in the frozen matrix at 77 K (Table 2); indeed, the intermediate rigidity of the polymeric matrix at 298 K partially inhibits the geometry relaxation of the emitting triplet states. On the contrary, in neat films, the blueshift of the emission bands of **1**–**3** is less pronounced and quantum yields are much lower and substantially comparable to room-temperature solution (Table 2). This is a commonly observed phenomenon in highly concentrated or pure films, where the complexes are close to each other and exciton diffusion becomes possible, leading to enhanced nonradiative pathways, as excited states encounter trapping sites.^78^ Also reabsorption and triplet–triplet quenching may play a role.

As already found in solution, a rather different scenario is observed for **4** and **5**. Indeed, since these complexes emit from strongly ligand-centered states, solvation/matrix effects are largely negligible and both emission spectra and quantum yields are virtually identical both in room-temperature solution and in solid state (Tables 2 and S7). Only a minor blueshift in the emission spectrum of **5** is observed in the PMMA matrix, probably due to limitations to excited-state distortions (Figure S32) induced by the polymeric matrix.

## Conclusions

Two triazole-based ligands have been designed to serve as cyclometalating agents by either C^∧^N (**A**) or C^∧^C: carbene (**B**) chelation. By combining these ligands with ancillary bipyridine-based or isocyanide ones, we obtained five iridium(III) complexes (**1**–**5**), with five fully distinct coordination environments, two of them as tris-heteroleptic systems (**2** and **5**). The electronic ground- and excited-state properties of **1**–**5** have been investigated by means of computational methods, electrochemistry and UV–vis absorption and emission spectroscopy. The HOMO–LUMO gap spans in the range of 2.57–3.86 eV, and the observed trends are fully rationalized based on the very peculiar coordination environment of every complex of the series. Luminescence bands of different nature (e.g., predominantly ^3^MLCT or ^3^LC) are observed all the way from blue to red and exhibit values of PLQY up to almost 60% in the PMMA matrix. The present work is a further step toward preparative routes of cyclometalated iridium(III) complexes that enable full control of every position of the octahedral coordination environment so that excited state and luminescence properties can be defined by design with yet a higher level of precision.

## Supporting Information Available

NMR spectra of ligands and complexes, X-ray data for ligand **B**, square-wave electrochemical data, TD-DFT excitations, triplet spin densities and energy diagrams, and experimental emission data (PDF)

## Accession Codes

CCDC 2153057 contains the supplementary crystallographic data for this paper. These data can be obtained free of charge via www.ccdc.cam.ac.uk/data_request/cif, or by emailing data_request@ccdc.cam.ac.uk, or by contacting The Cambridge Crystallographic Data Centre, 12 Union Road, Cambridge CB2 1EZ, UK; fax: +44 1223 336033.
