# Supplementary material for: 4-Phenyl-1,2,3-triazoles as Versatile Ligands for Cationic Cyclometalated Iridium(III) Complexes
Source: Inorg Chem. 2022 May 24;61(22):8509–20. doi: 10.1021/acs.inorgchem.2c00567 (PMC9490865; doi:10.1021/acs.inorgchem.2c00567)
Supplement: Supplementary file 1 — ic2c00567_si_001.pdf [file ic2c00567_si_001.pdf]

Supporting Information

4-Phenyl-1,2,3-triazoles as Versatile Ligands for Cationic Cyclometalated Iridium(III) Complexes

Alessandro Di Girolamo,[§] Filippo Monti,*[†] Andrea Mazzanti,[§] Elia Matteucci,[§] Nicola Armaroli,[†] Letizia Sambri,[§] and Andrea Baschieri*[†]

§ Department of Industrial Chemistry "Toso Montanari", University of Bologna, Bologna 40136, Italy

† Istituto per la Sintesi Organica e la Fotoreattività, Consiglio Nazionale delle Ricerche, Bologna 40129, Italy

E-mail: F. M.: filippo.monti@isof.cnr.it
A. B.: andrea.baschieri@isof.cnr.it

Table of Contents

Contents	*Pages*
NMR spectra of ligands **A** and **B**	S2 – S5
NMR spectrum of ligand **B** deprotonated	S6
NMR spectra of complexes **1**–**5**	S7 – S18
ESI$^+$ spectrum of complex **2**	S18
Orbital-interaction diagram for **1** and **3**	S19
Electrochemical data	S20
Absorption spectra of **1**–**5** in CH_2Cl_2	S21
TD-DFT vertical excitations for **1**–**5**	S22 – S26
U-DFT emission and excited-state data	S27 – S28
X-ray data for compound **B**	S29 – S33

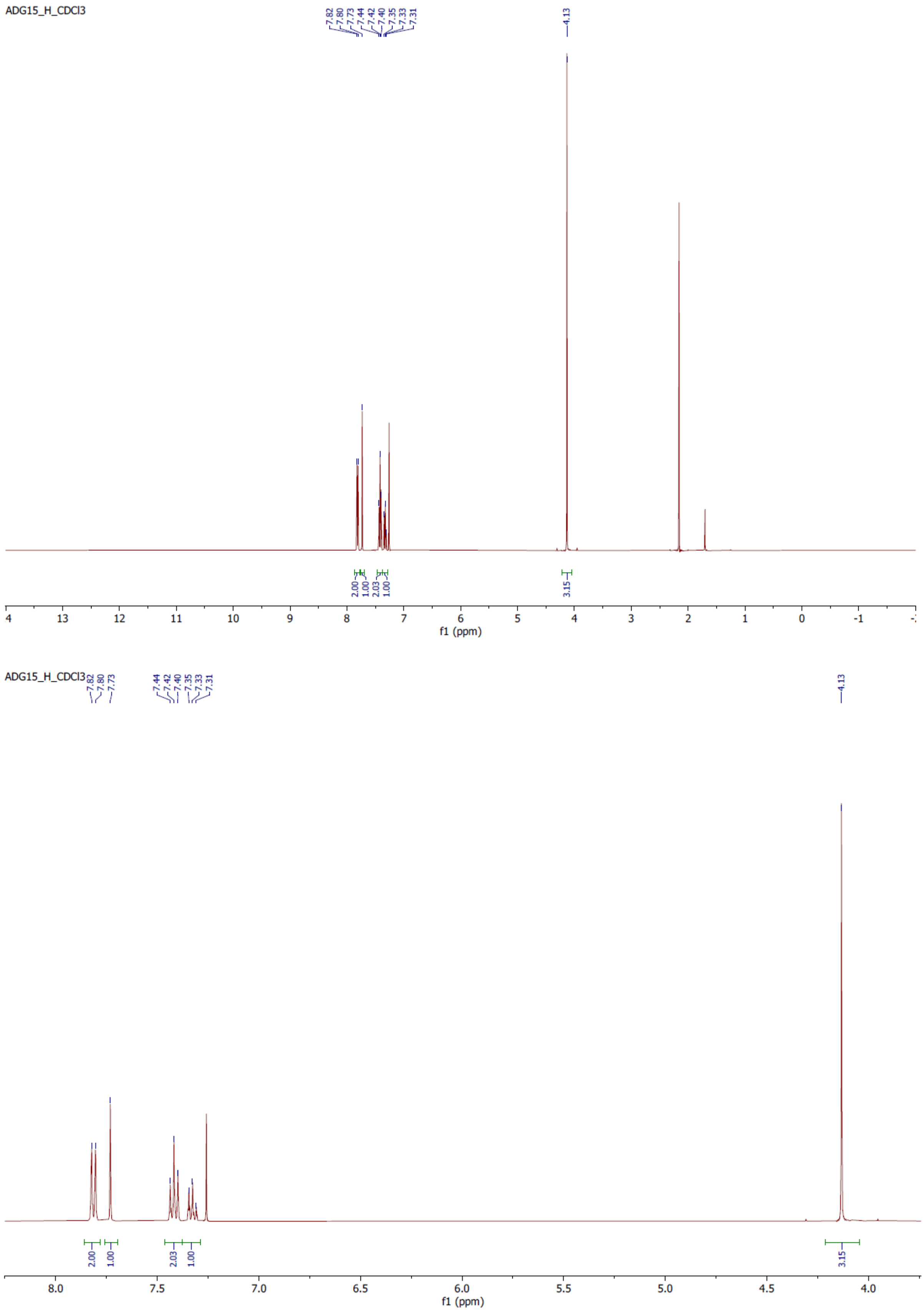

Figure S1. ^{1}H NMR spectrum of ligand **A**.

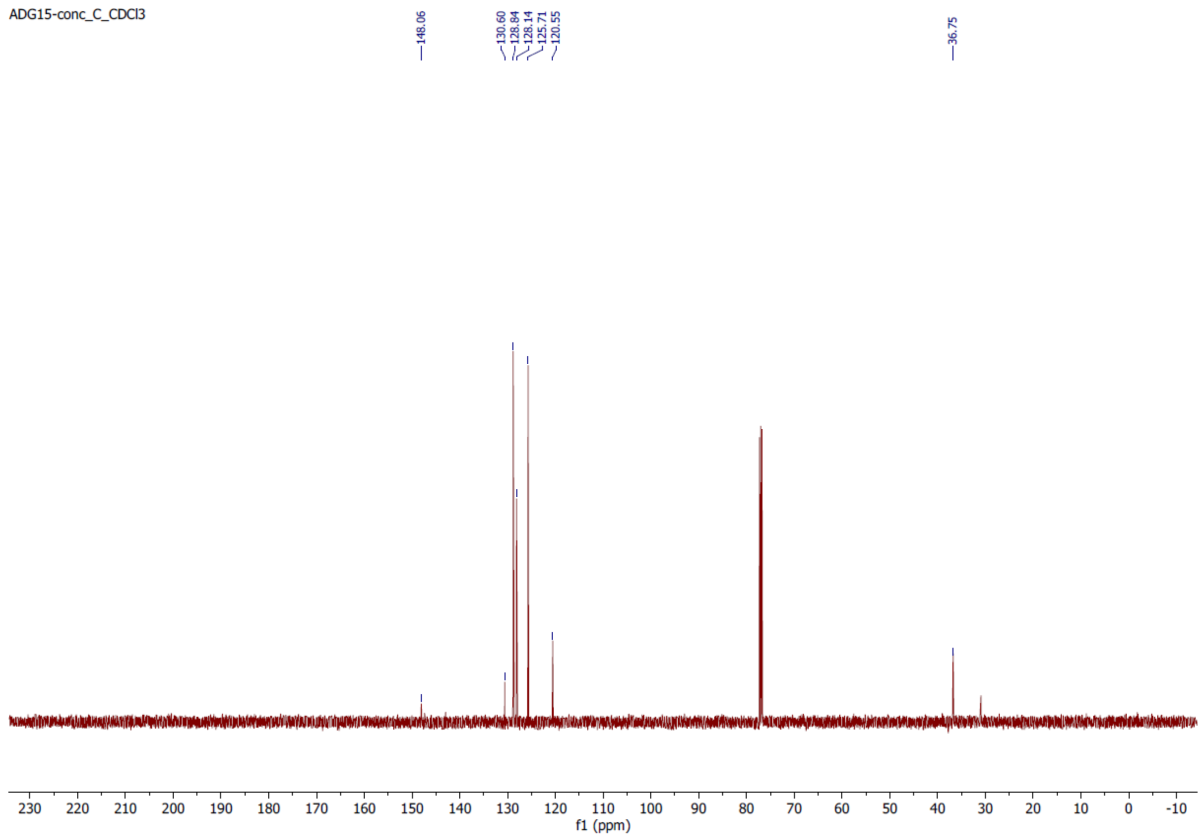

Figure S2. ^{13}C NMR spectrum of ligand **A**.

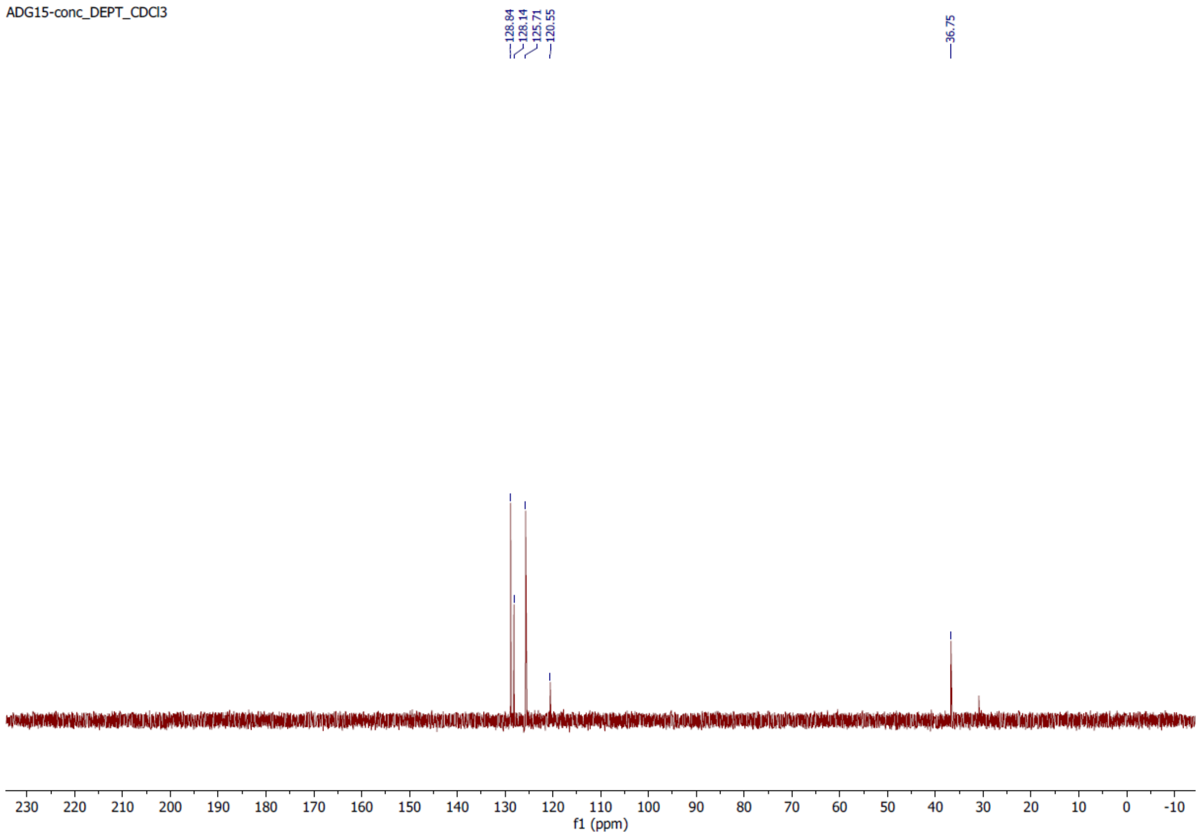

Figure S3. DEPT 135 NMR spectrum of ligand **A**.

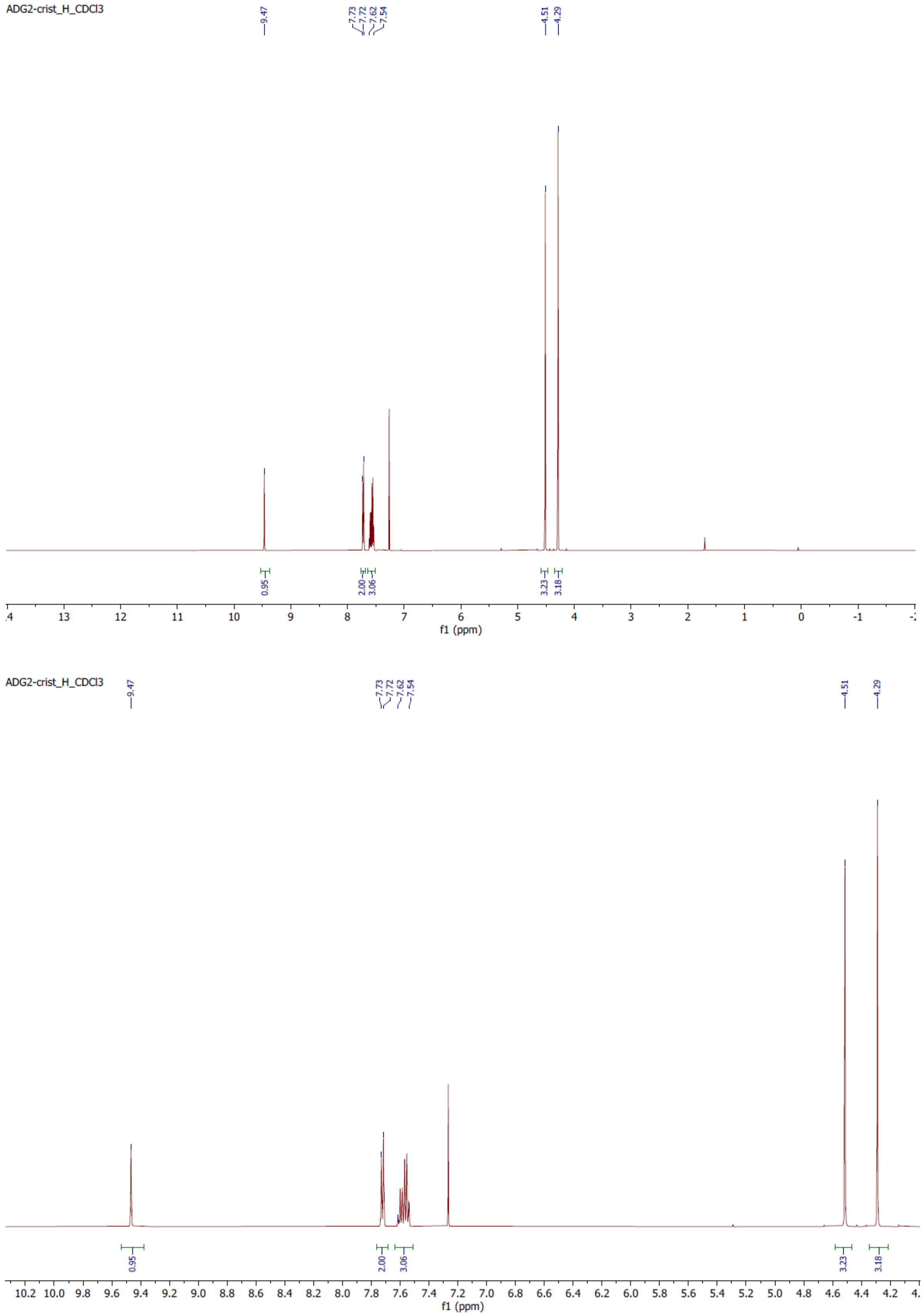

Figure S4. ^{1}H NMR spectrum of ligand **B**.

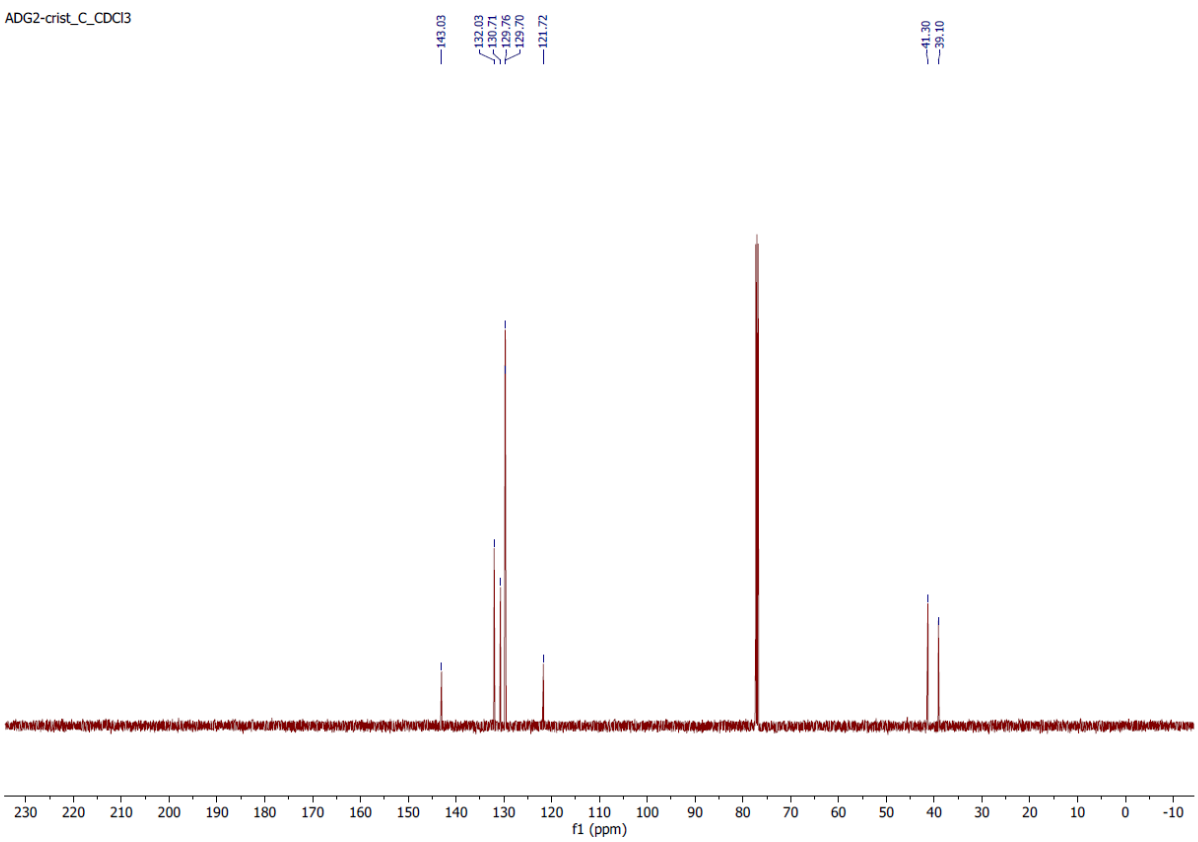

Figure S5. ^{13}C NMR spectrum of ligand **B**.

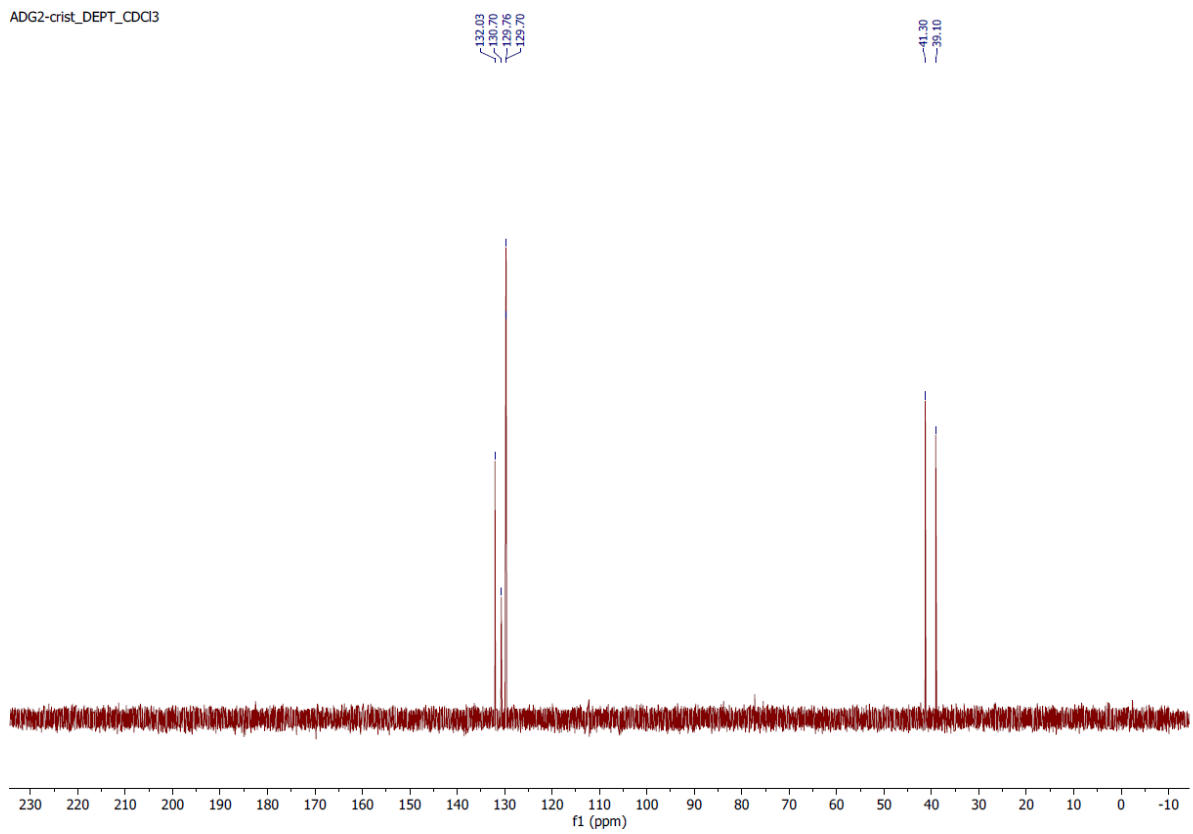

Figure S6. DEPT 135 NMR spectrum of ligand **B**.

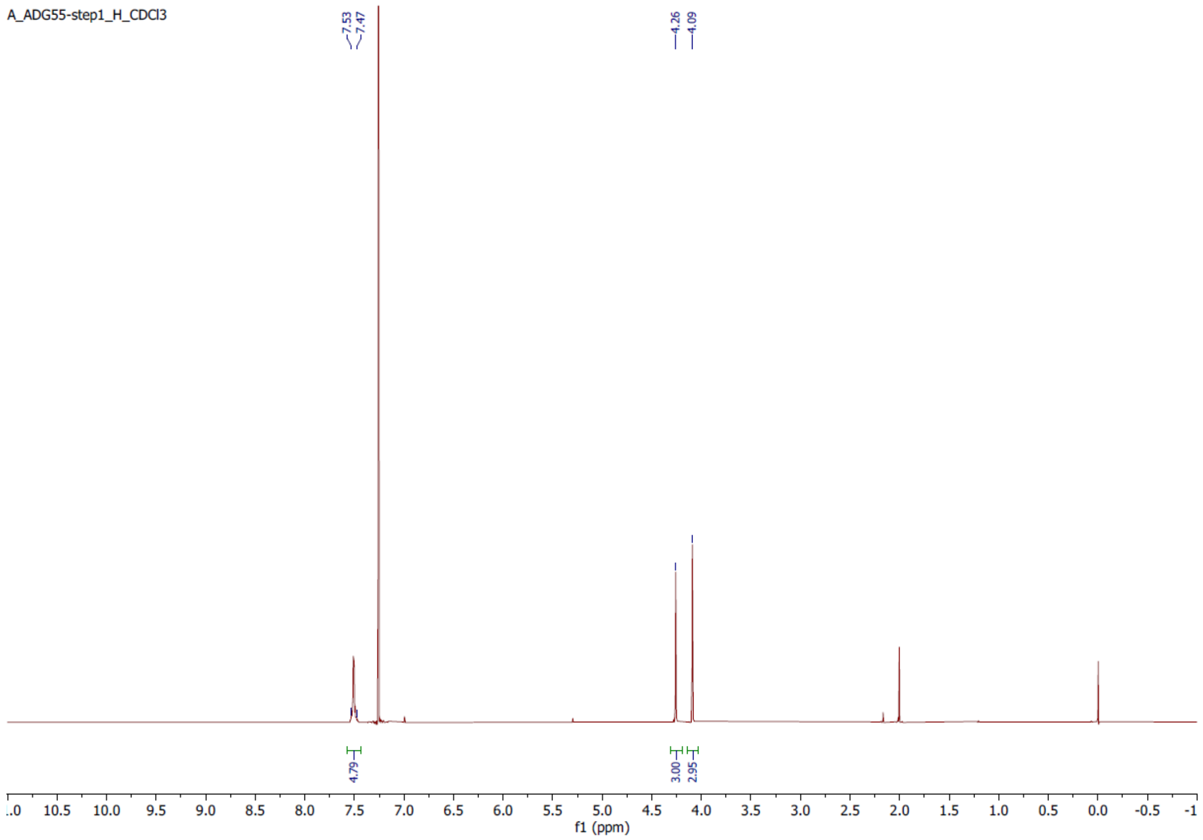

Figure S7. ^{1}H NMR spectrum of ligand **B** deprotonated.

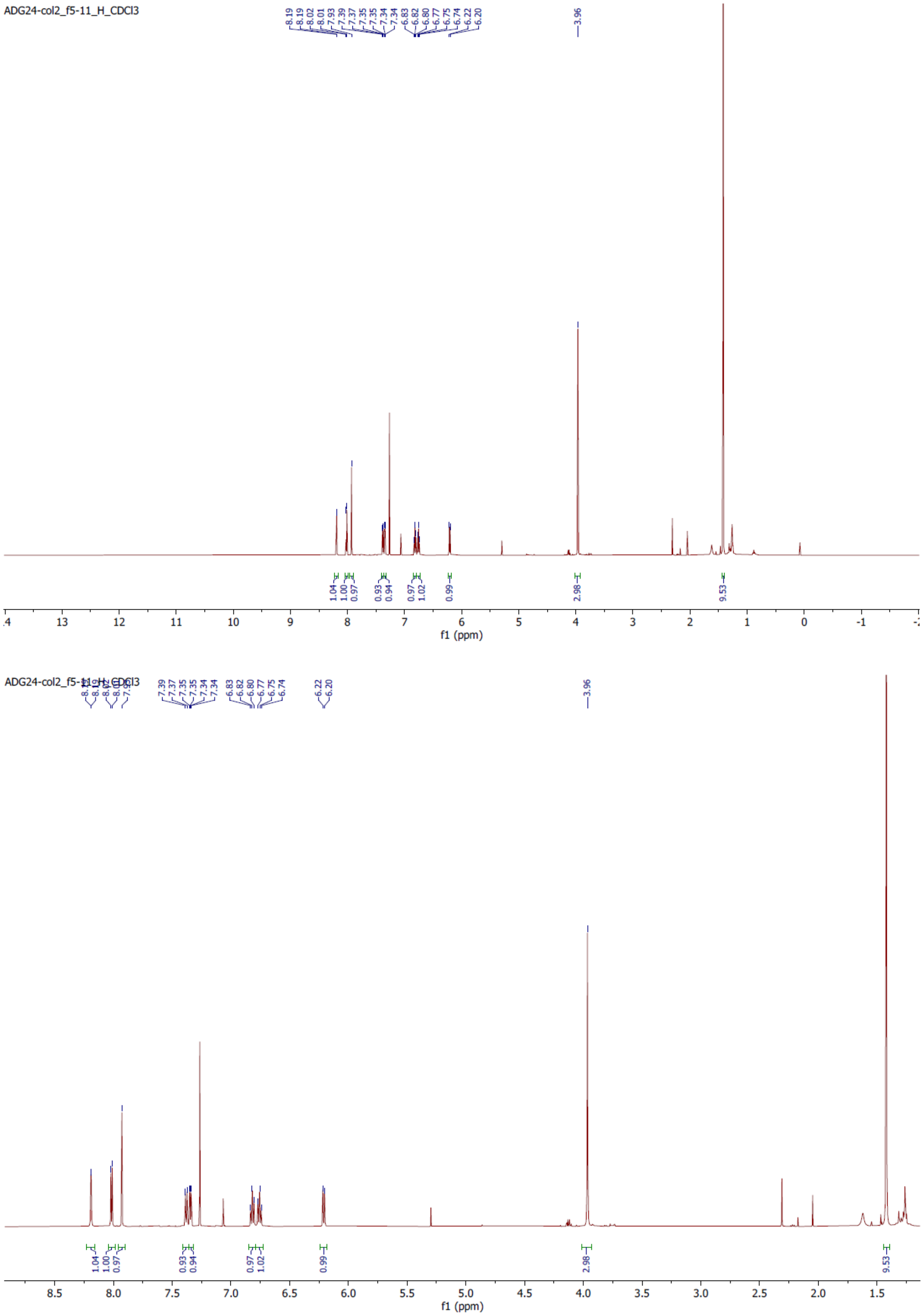

Figure S8. ^{1}H NMR spectrum of complex **1**.

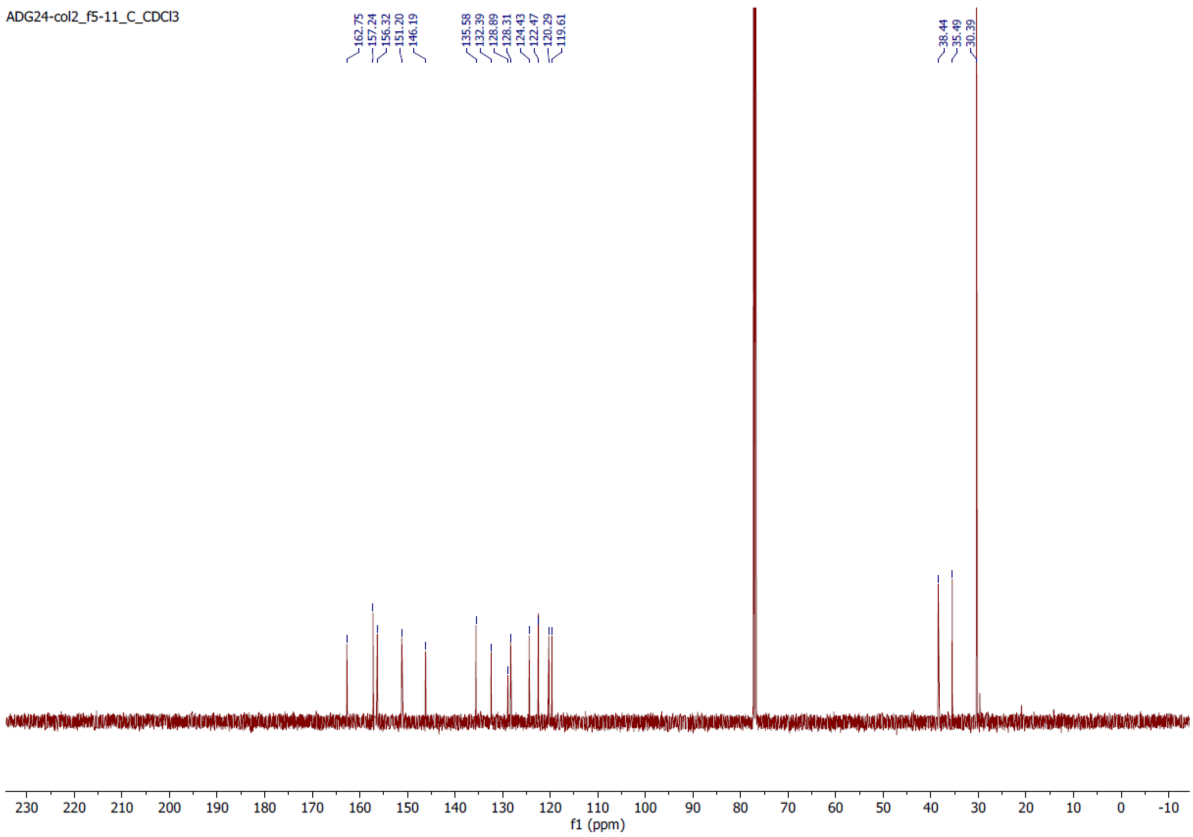

Figure S9. ^{13}C NMR spectrum of complex **1**.

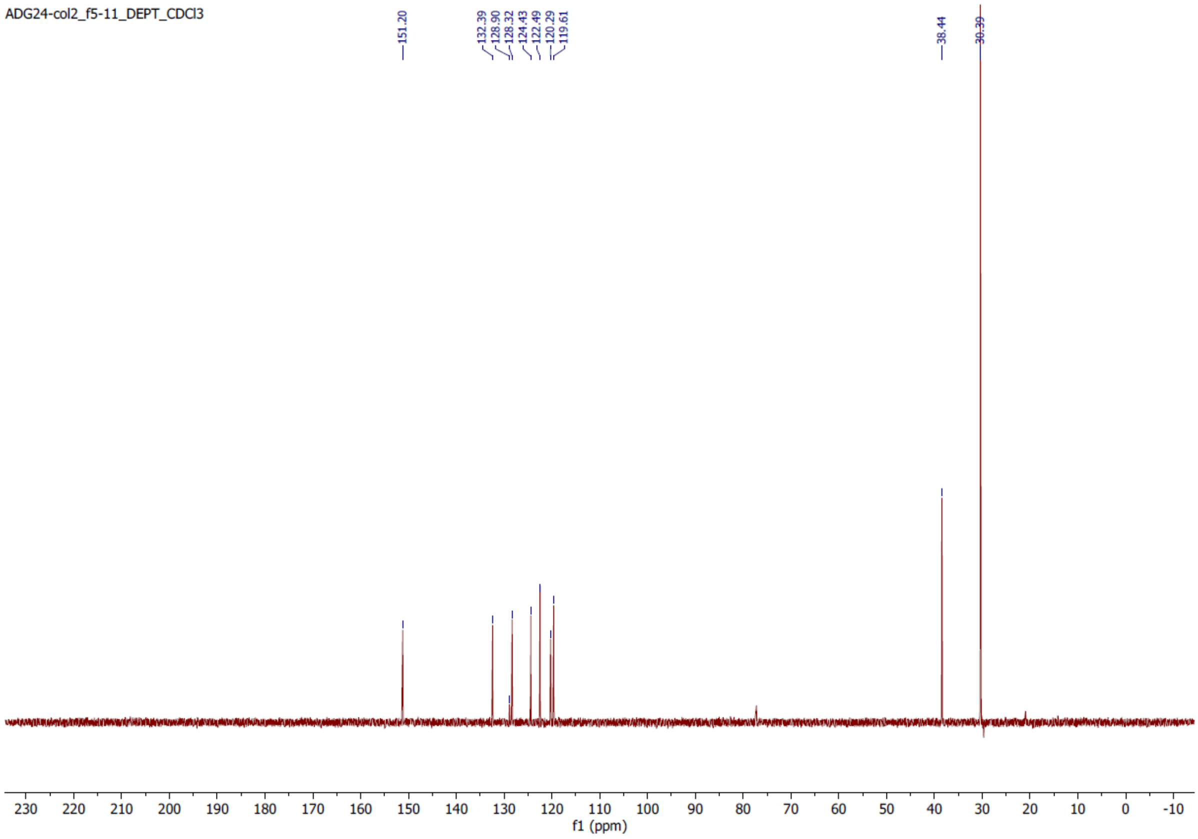

Figure S10. DEPT 135 NMR spectrum of complex **1**.

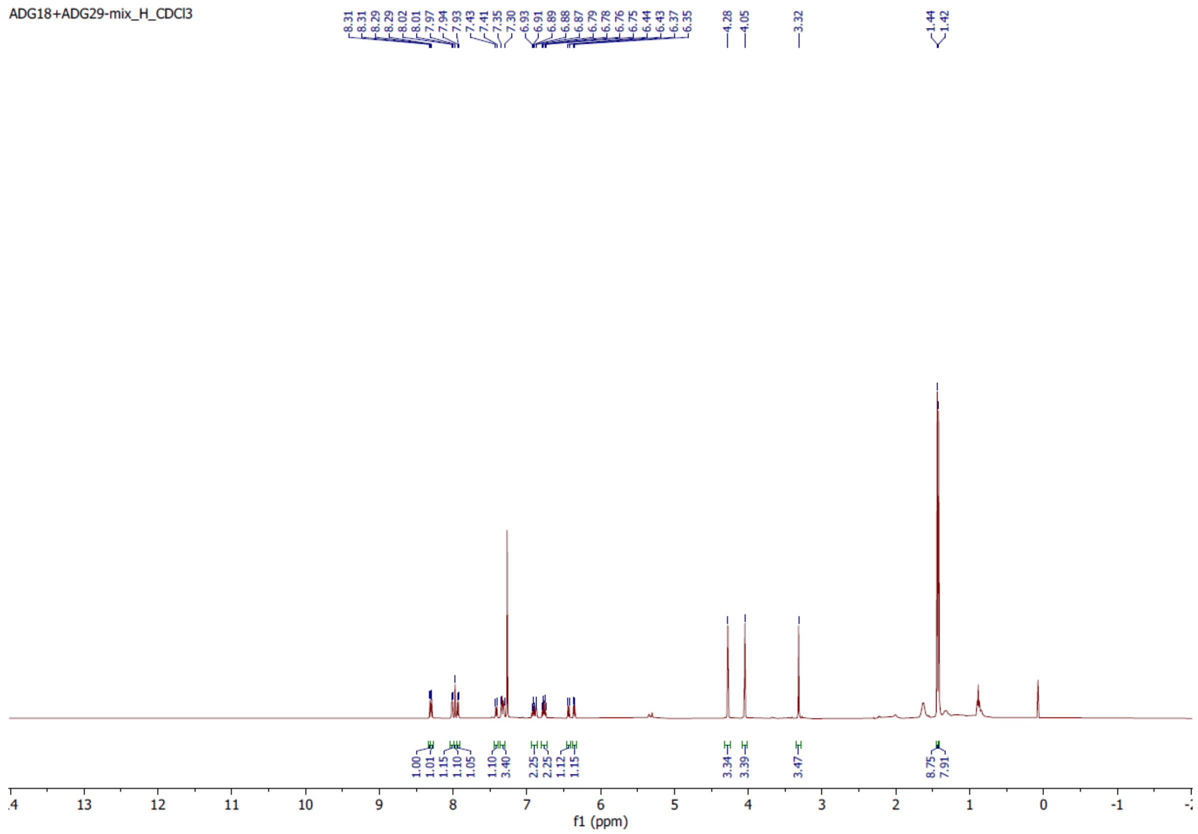

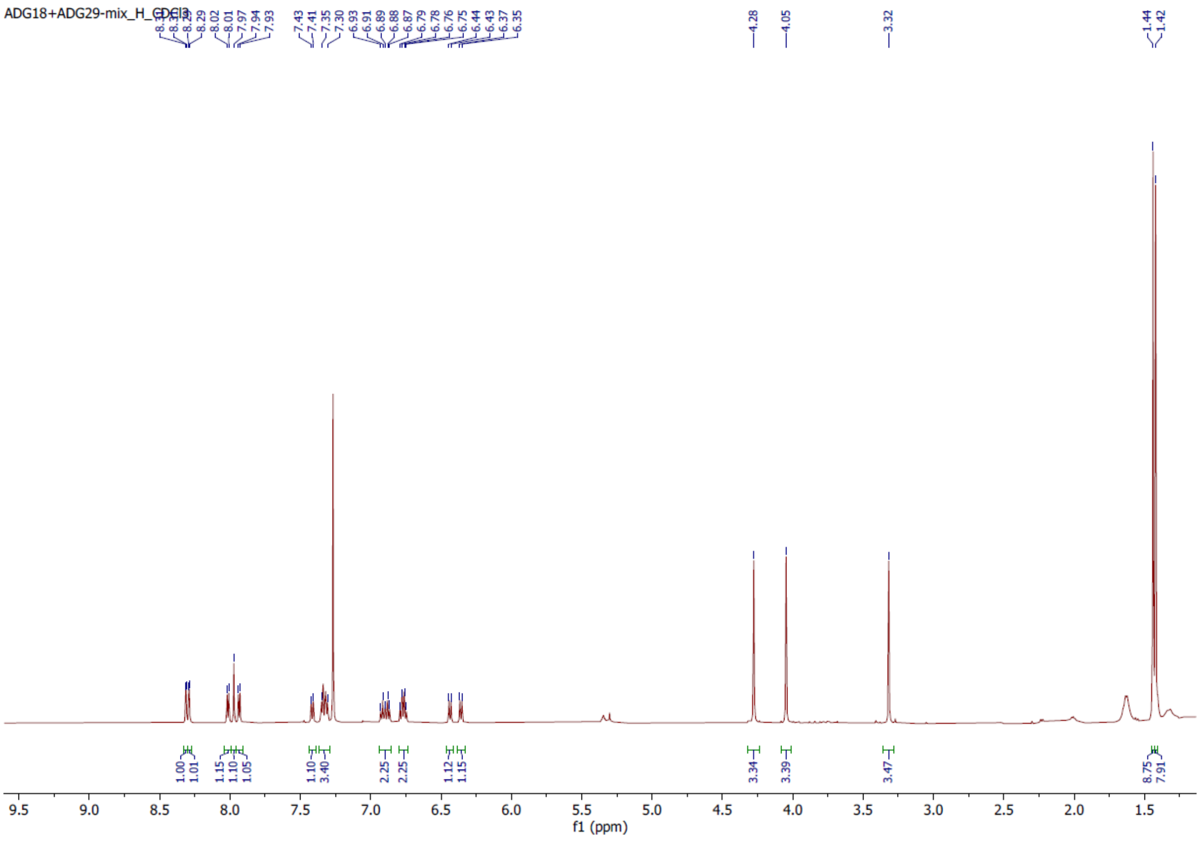

Figure S11. ^{1}H NMR spectrum of complex **2**.

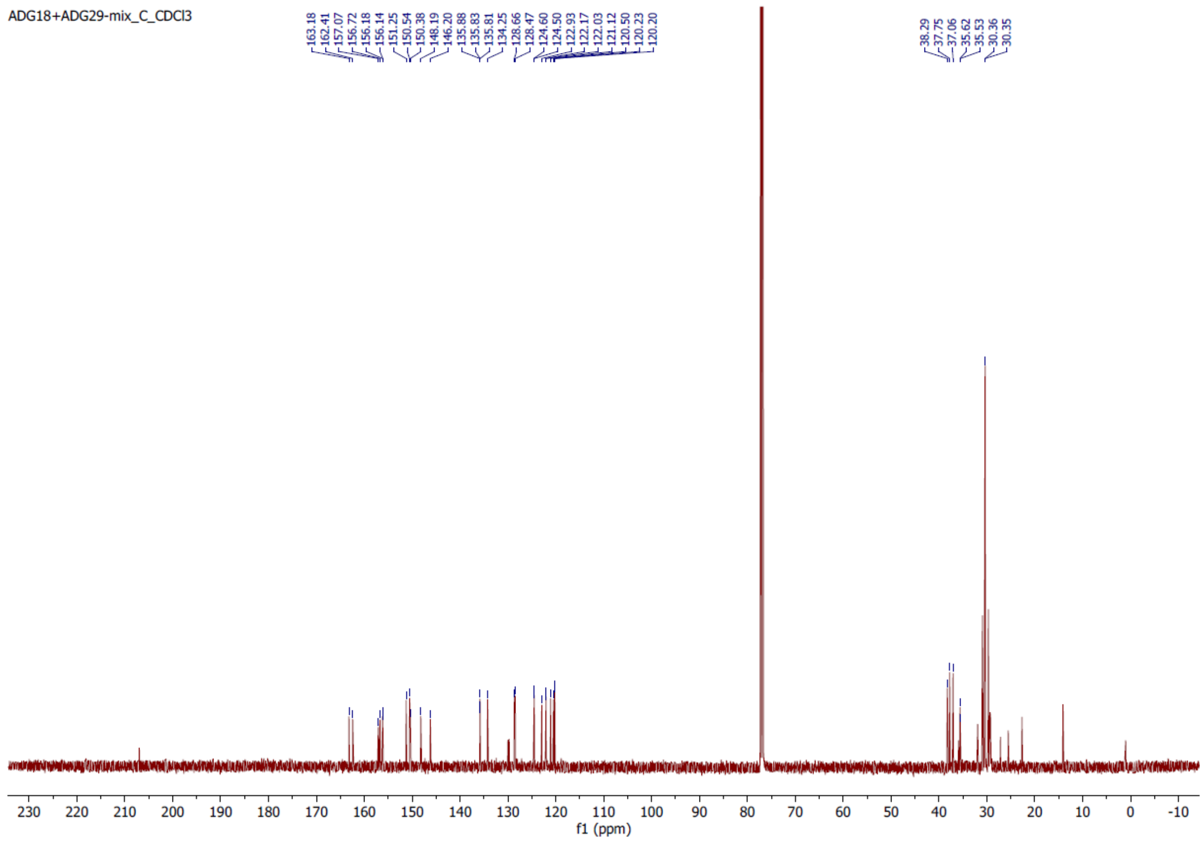

Figure S12. ^{13}C NMR spectrum of complex **2**.

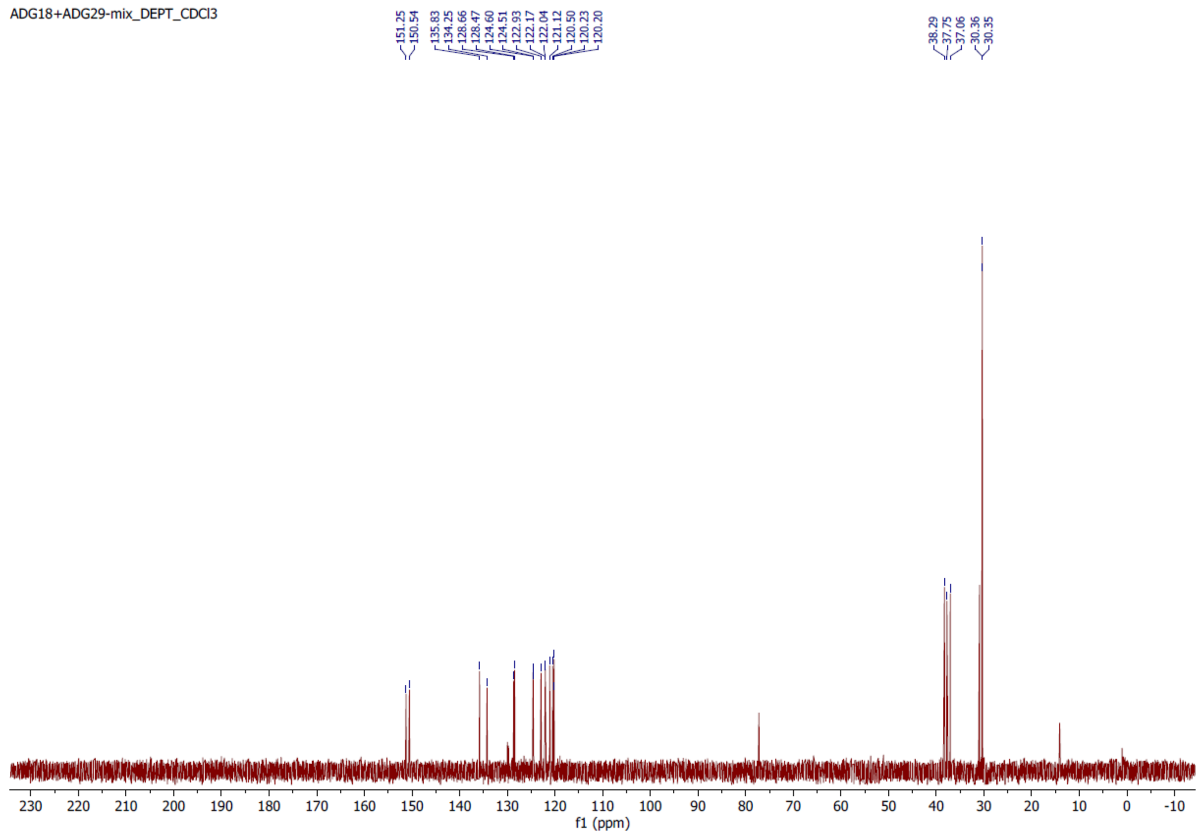

Figure S13. DEPT 135 NMR spectrum of complex **2**.

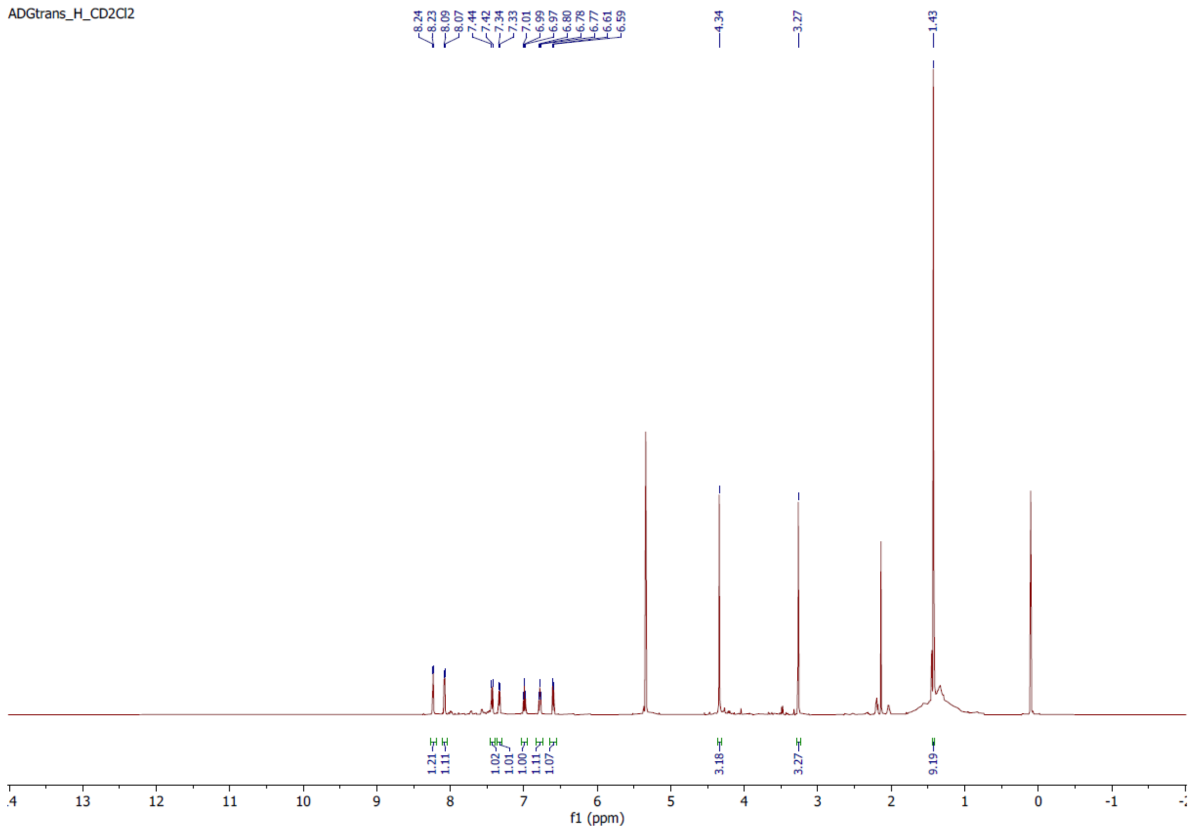

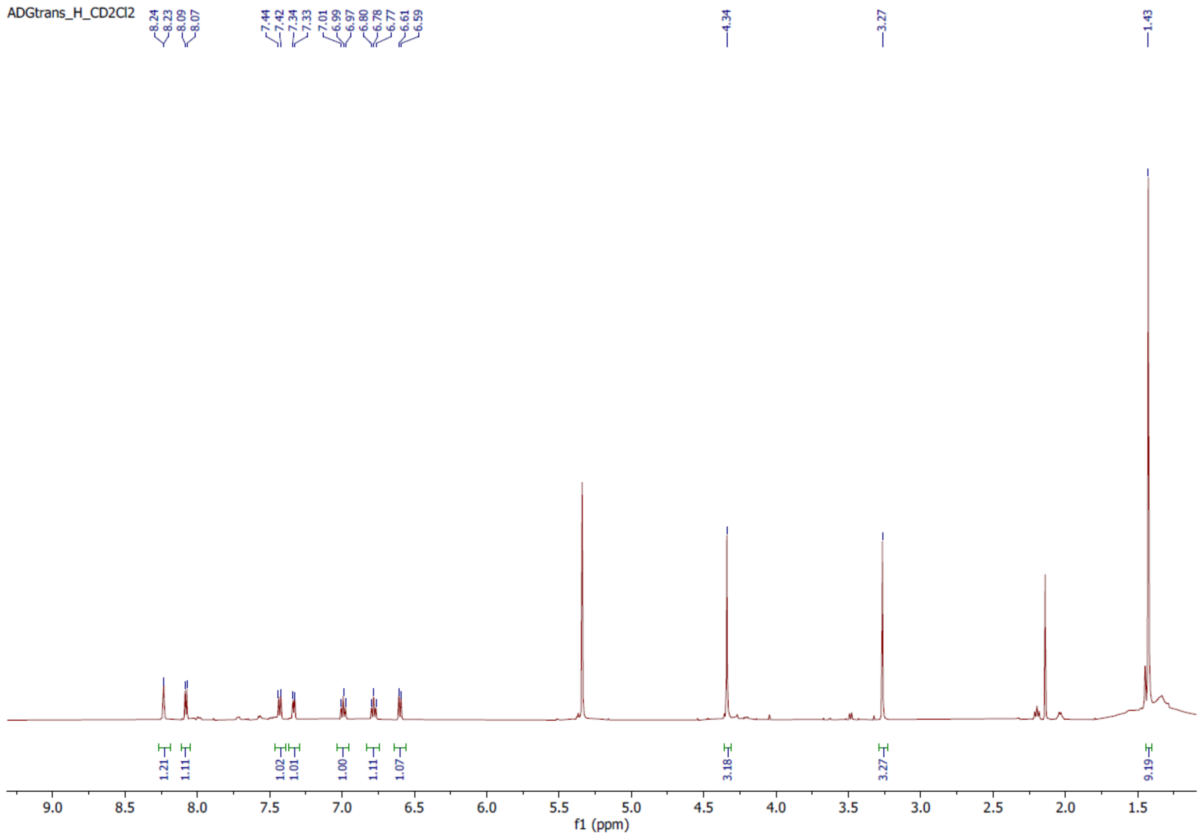

Figure S14. ^{1}H NMR spectrum of complex **3**.

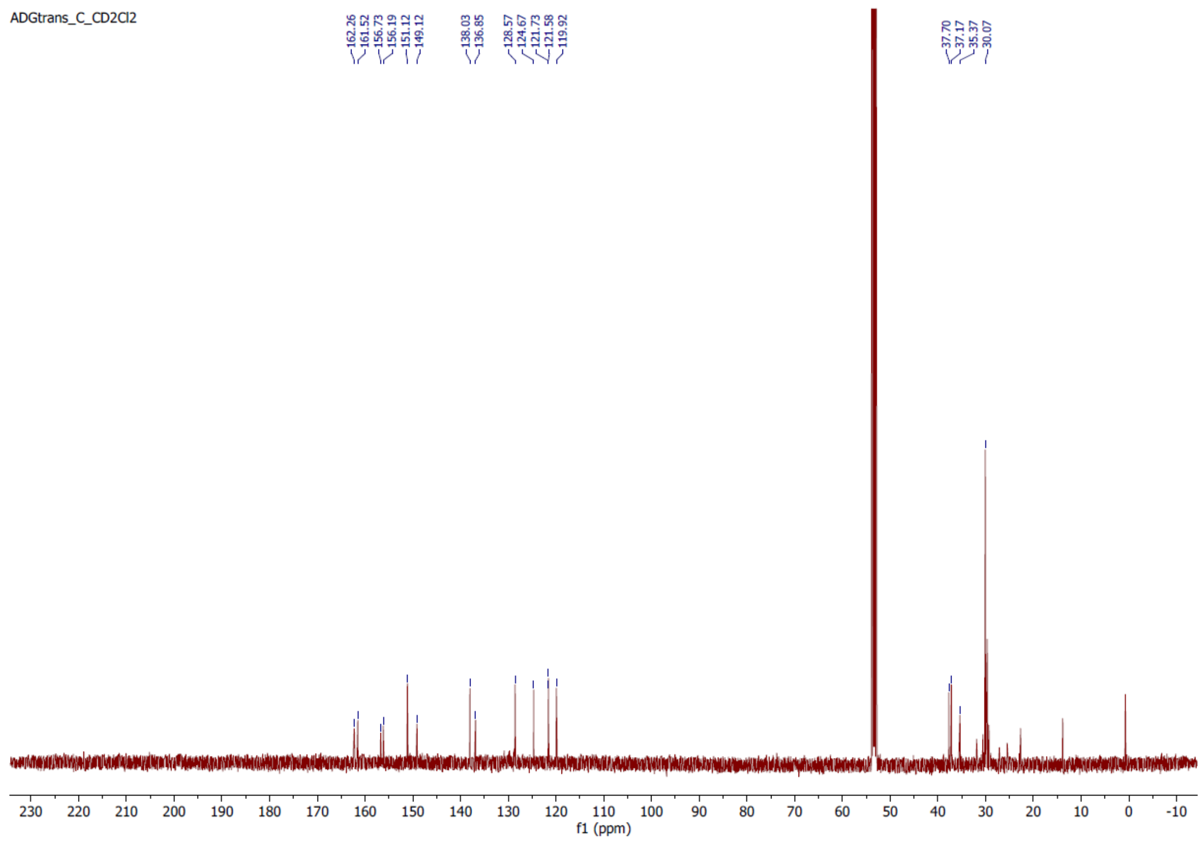

Figure S15. ^{13}C NMR spectrum of complex **3**.

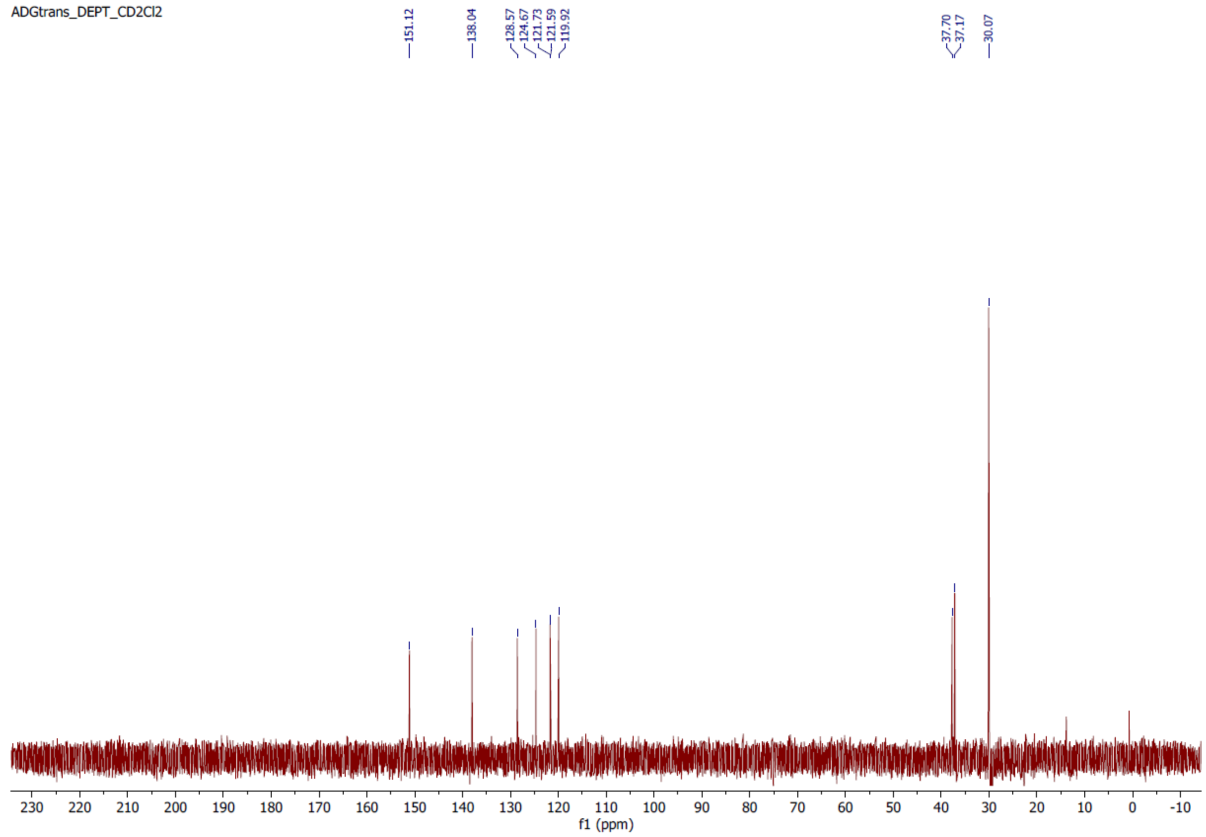

Figure S16. DEPT 135 NMR spectrum of complex **3**.

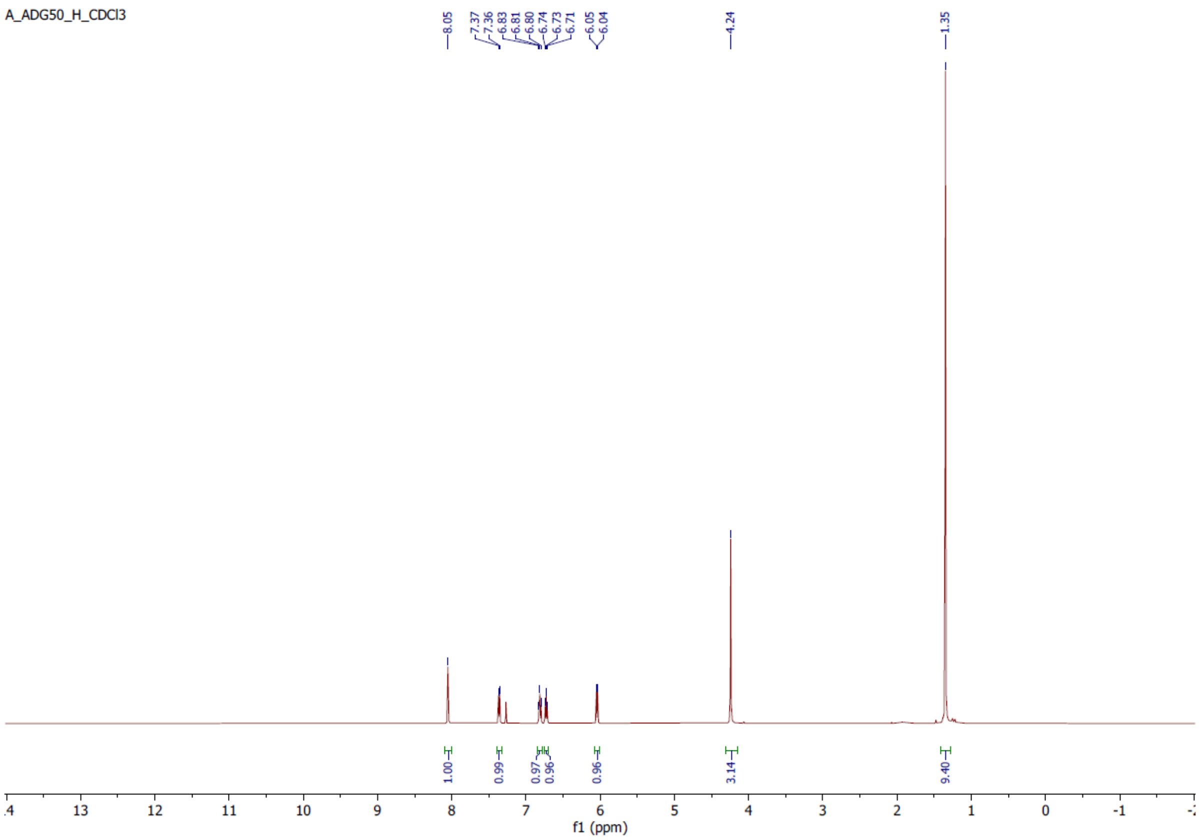

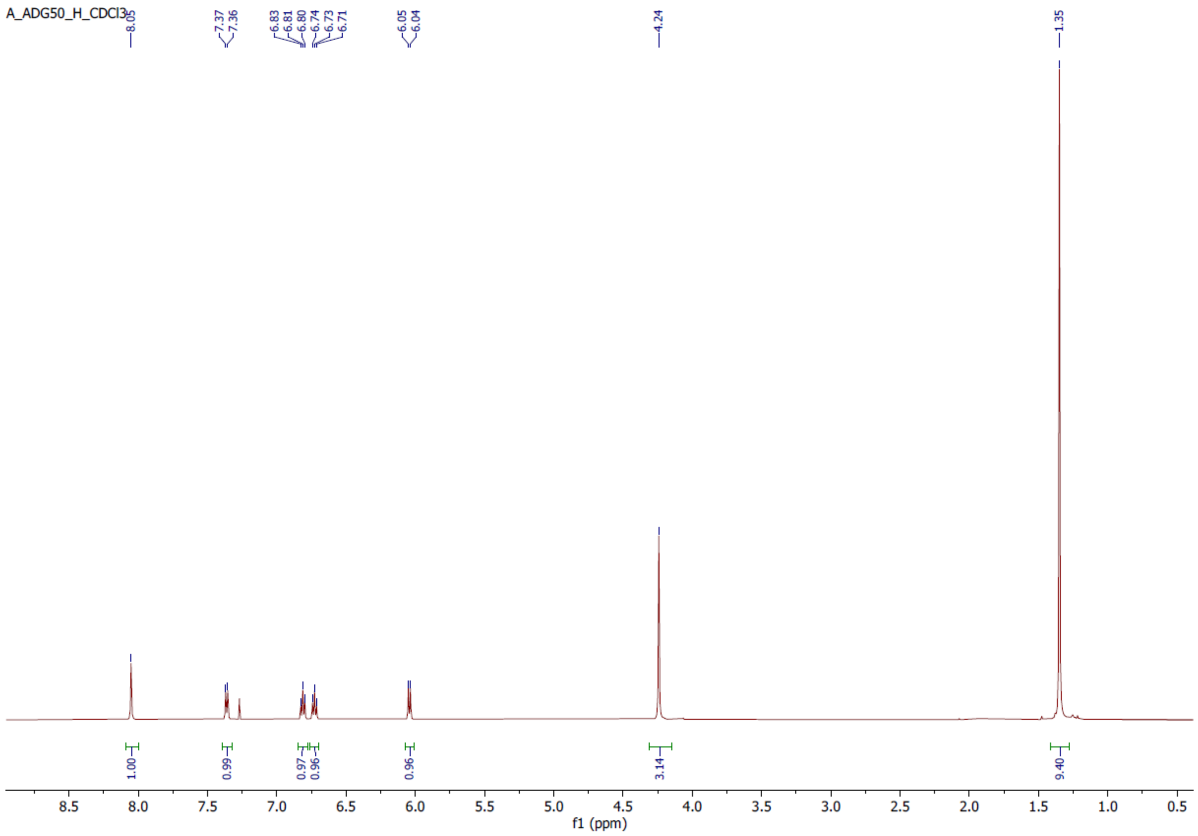

Figure S17. ^{1}H NMR spectrum of complex **4**.

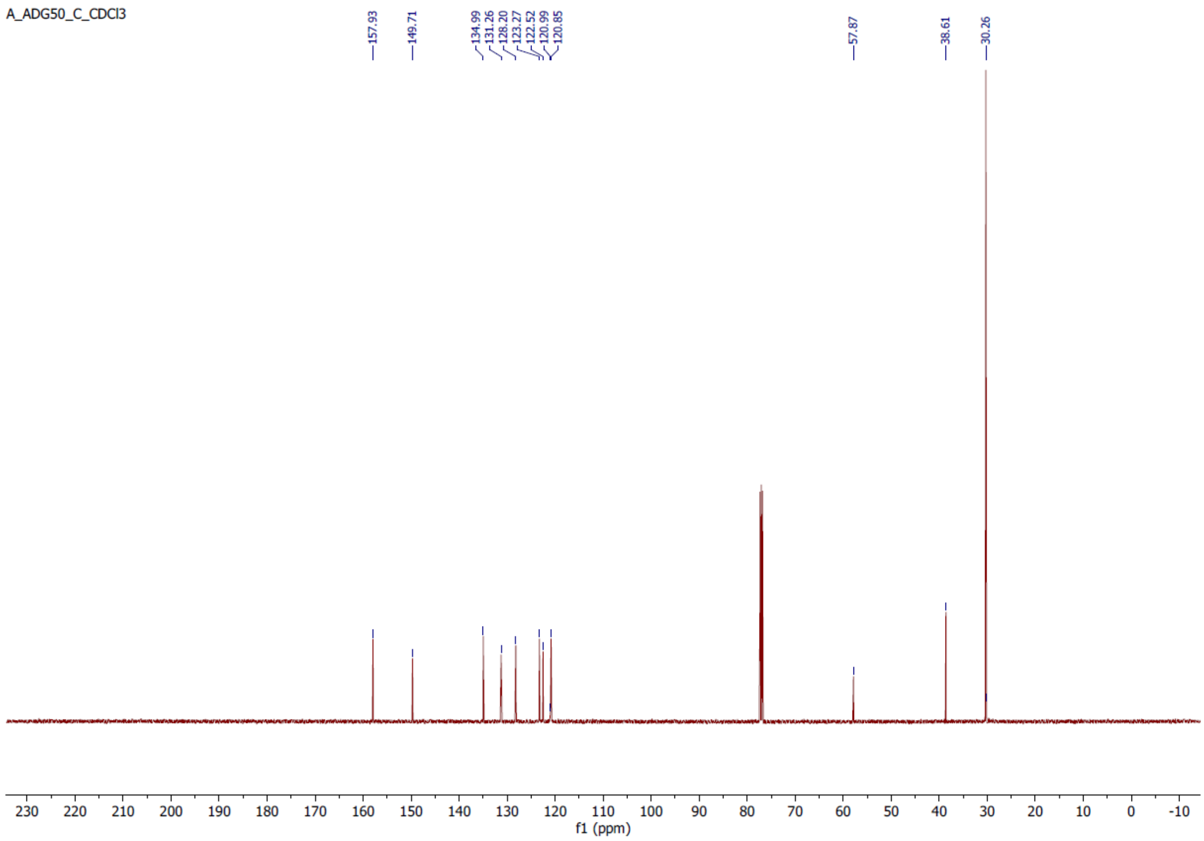

Figure S18. ^{13}C NMR spectrum of complex **4**.

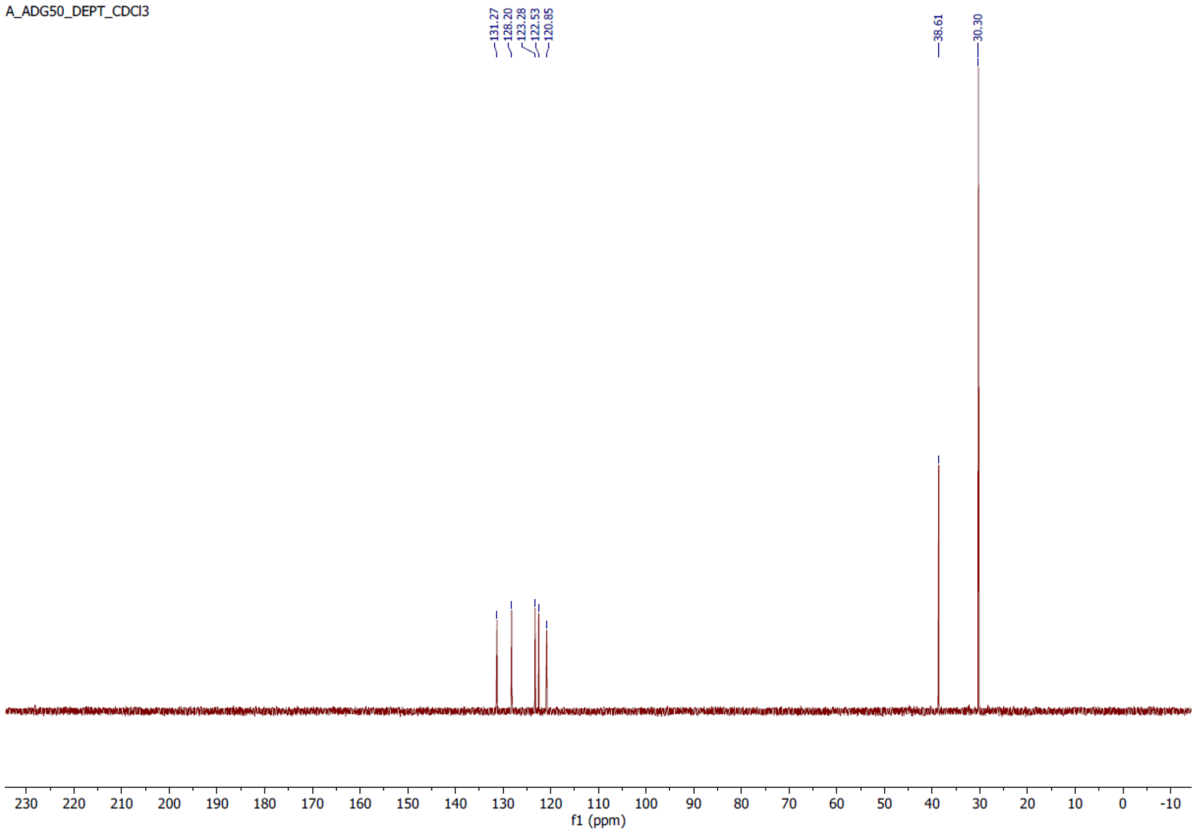

Figure S19. DEPT 135 NMR spectrum of complex **4**.

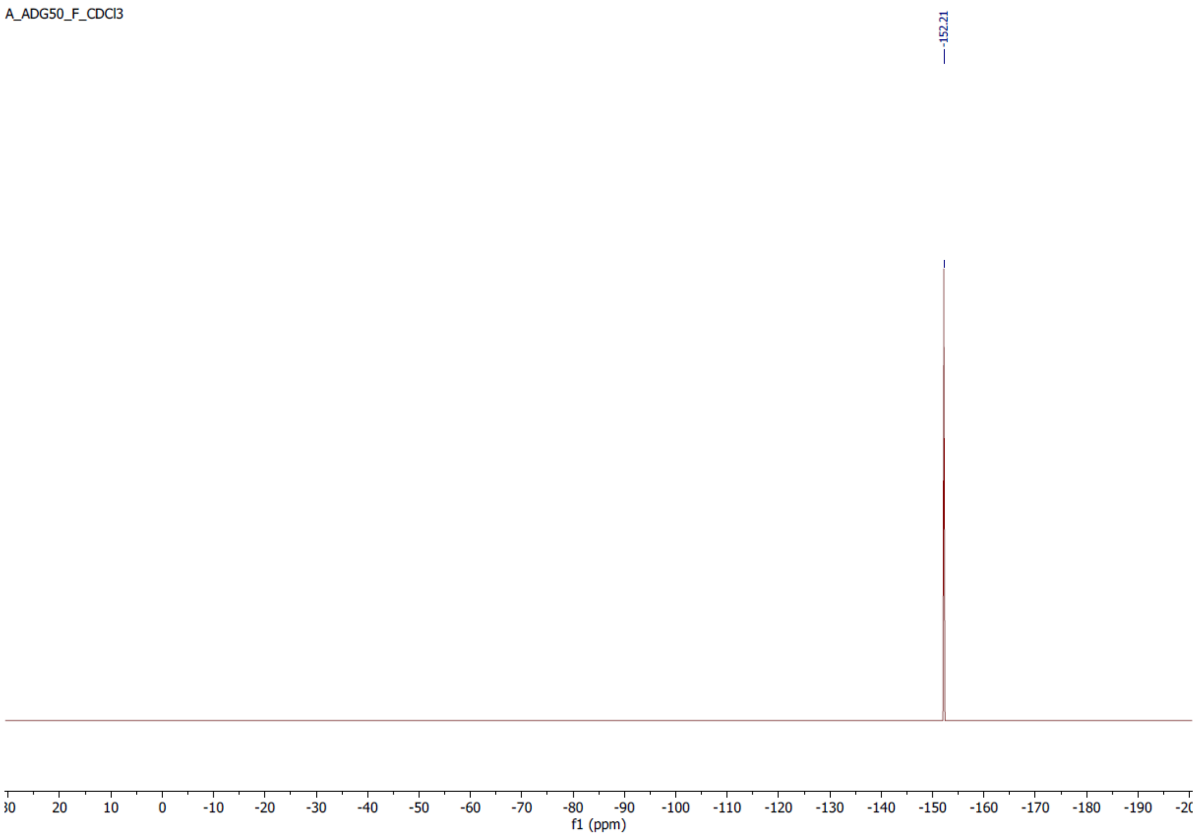

Figure S20. ^{19}F NMR spectrum of complex **4**.

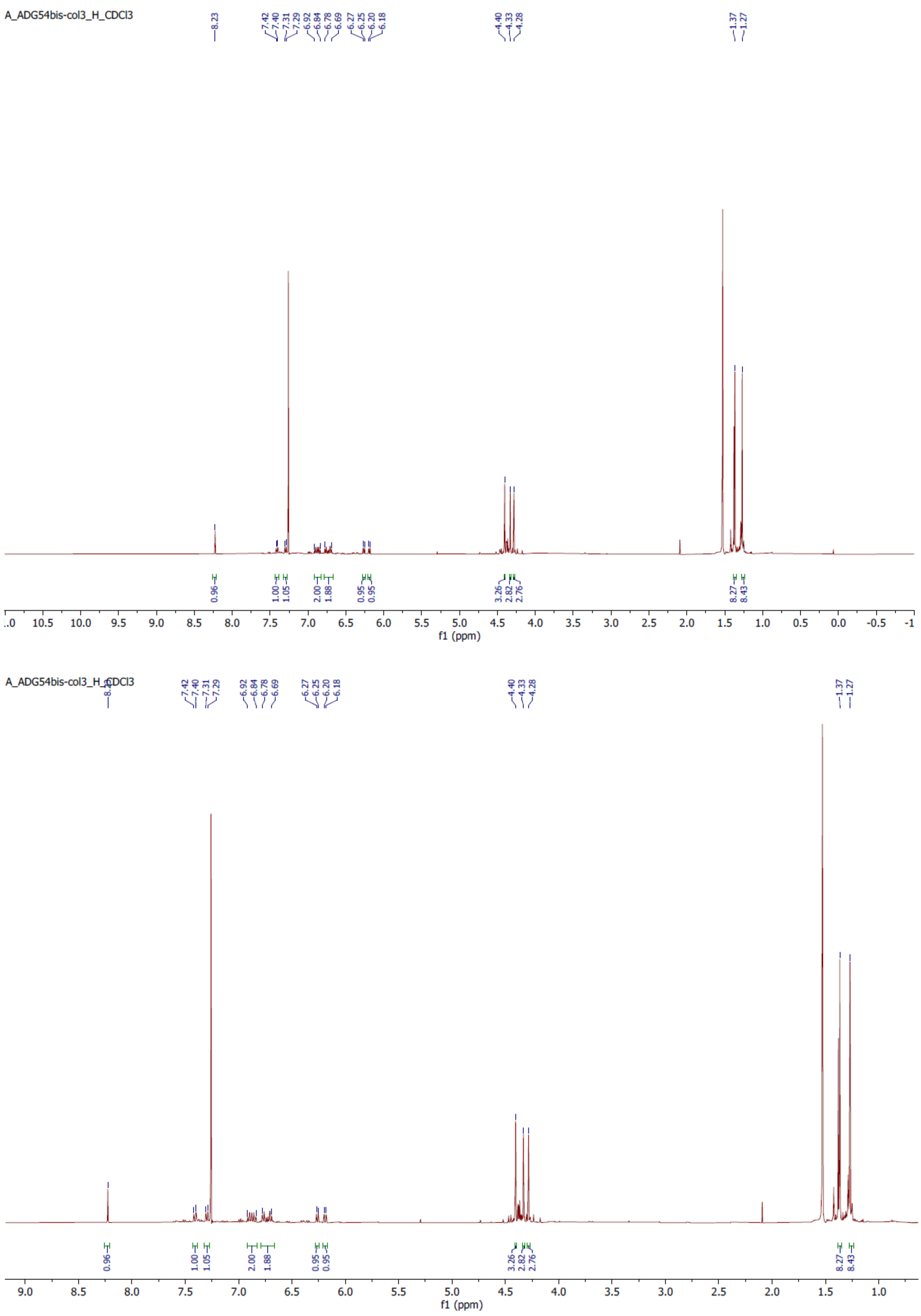

Figure S21. ^{1}H NMR spectrum of complex **5**.

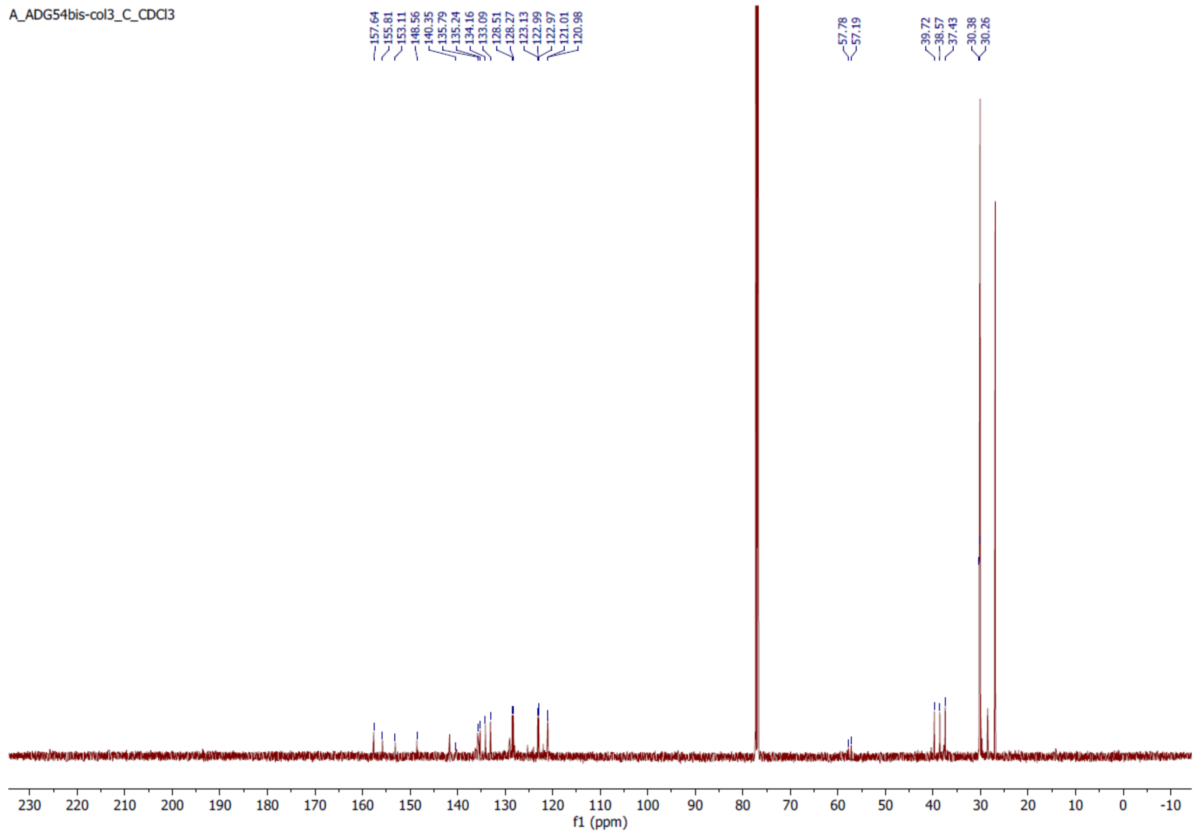

Figure S22. ^{13}C NMR spectrum of complex **5**.

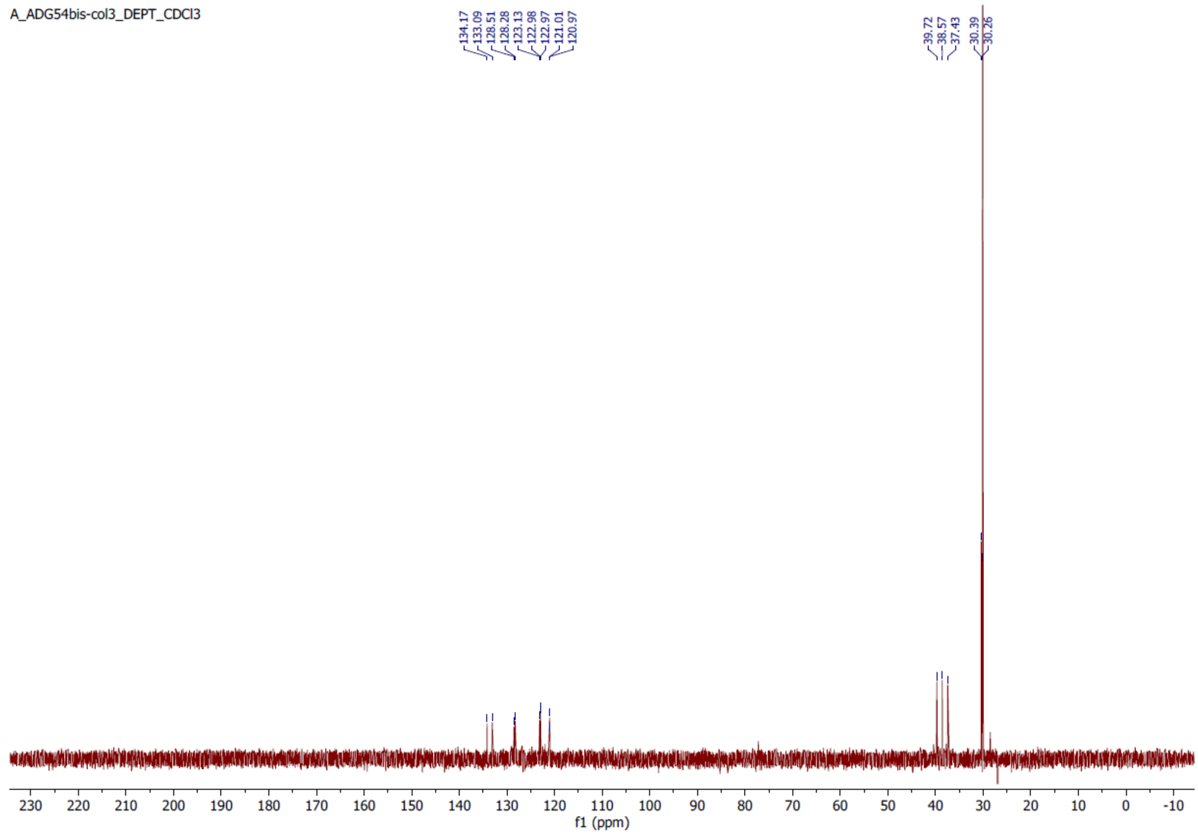

Figure S23. DEPT 135 NMR spectrum of complex **5**.

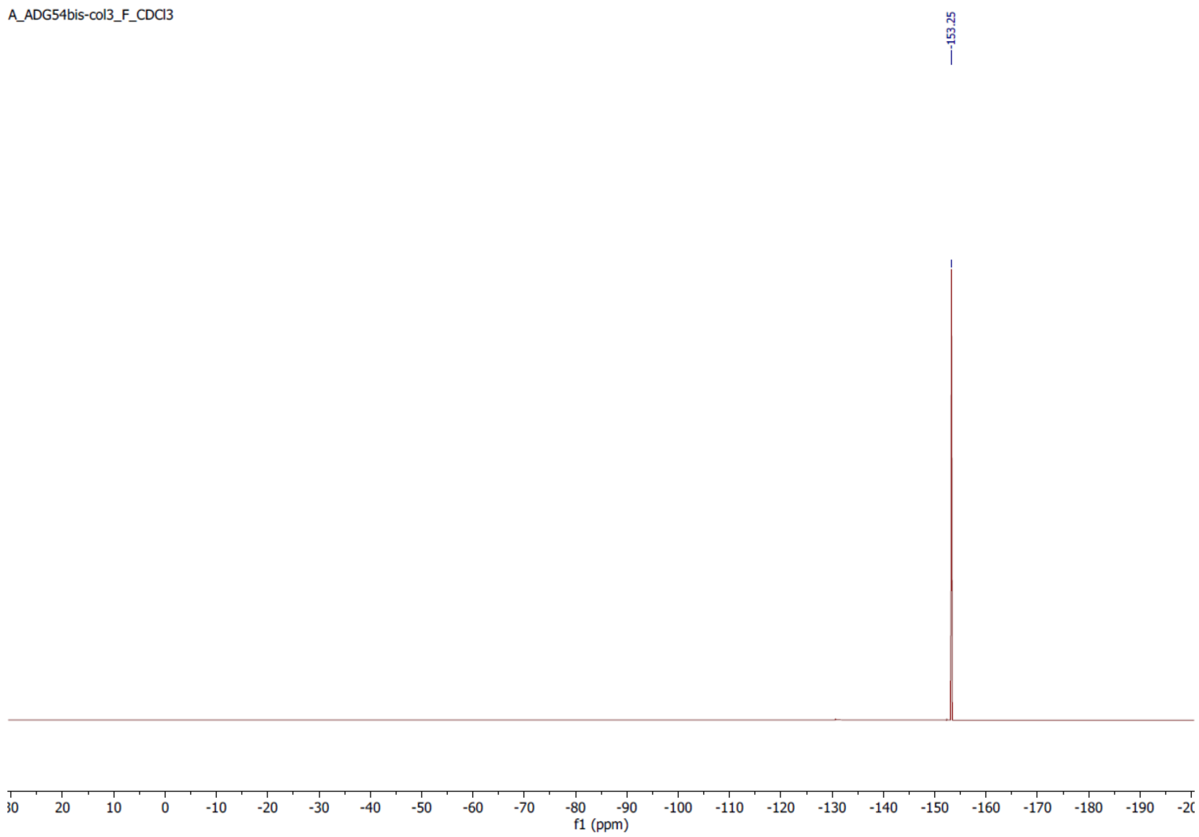

Figure S24. ^{19}F NMR spectrum of complex **5**.

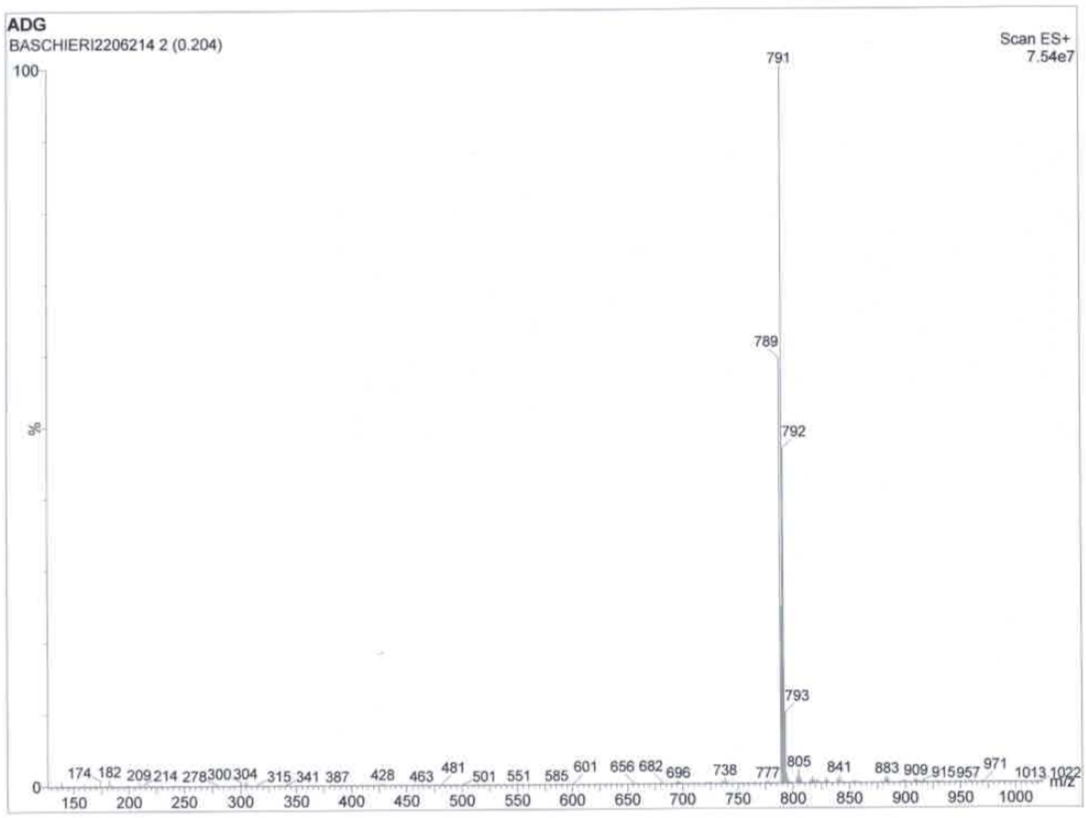

Figure S25. ESI^{+} spectrum of complex **2**.

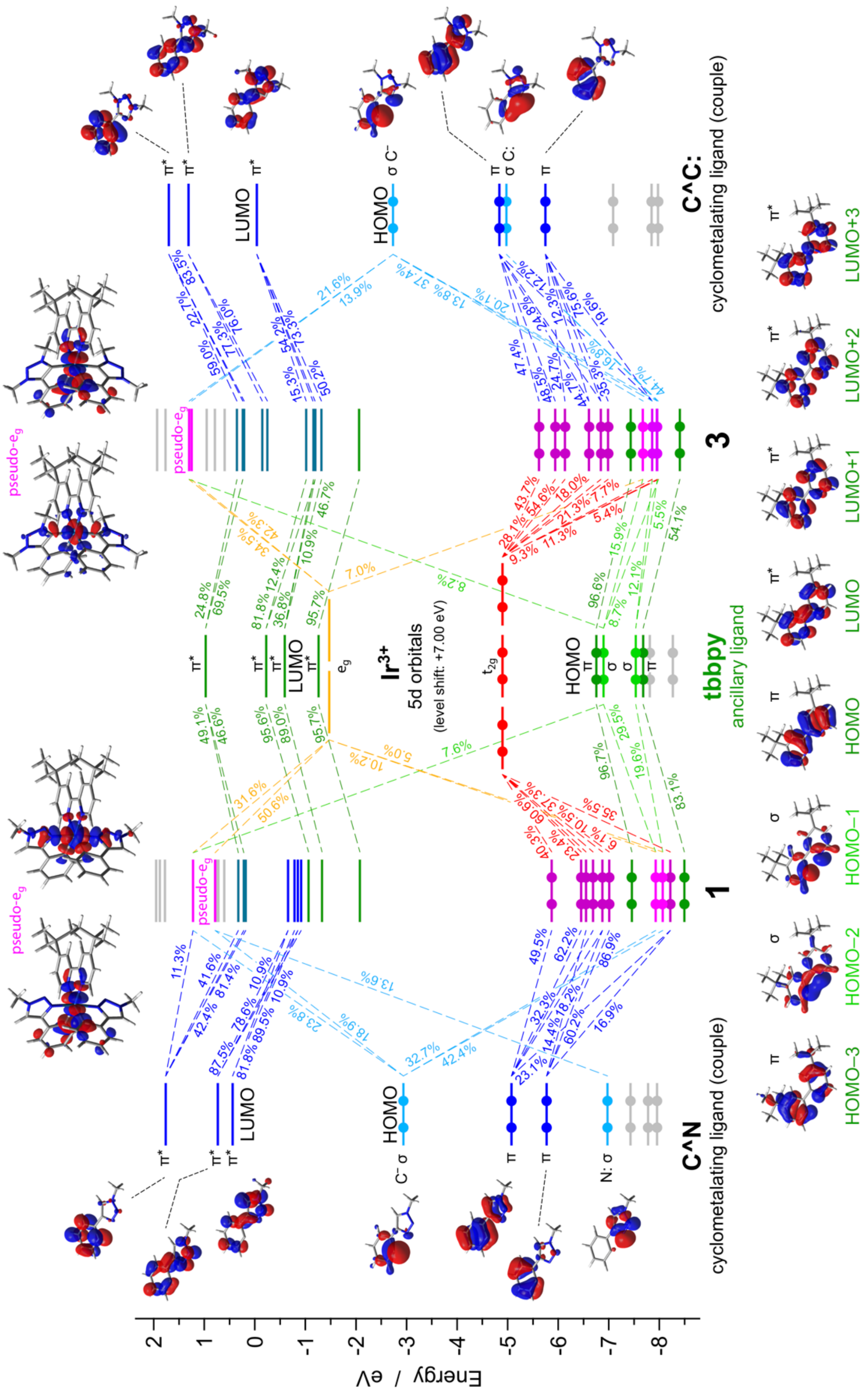

Figure S26. Orbital-interaction diagram of complexes **1** and **3**, calculated in acetonitrile using charge decomposition analysis (see Experimental Section for further details). Fragment orbitals are computed by dividing each complex into 4 fragments: the Ir^{3+} central ion, the two degenerate anionic cyclometalating ligands (C^N or C^C:) and the neutral 4,4'-di-*tert* butyl-2,2'-bipyridine ancillary ligand (dtbbpy). Only fragment-orbital contributions above 5.0% are reported. A level shift of +7.00 eV is applied to the Ir^{3+} fragment for a better graph visualization. Orbitals colored in light gray are not analyzed since not contributing to the depicted complex frontier orbitals (ligand case) or because having extremely diffuse character (complex case).

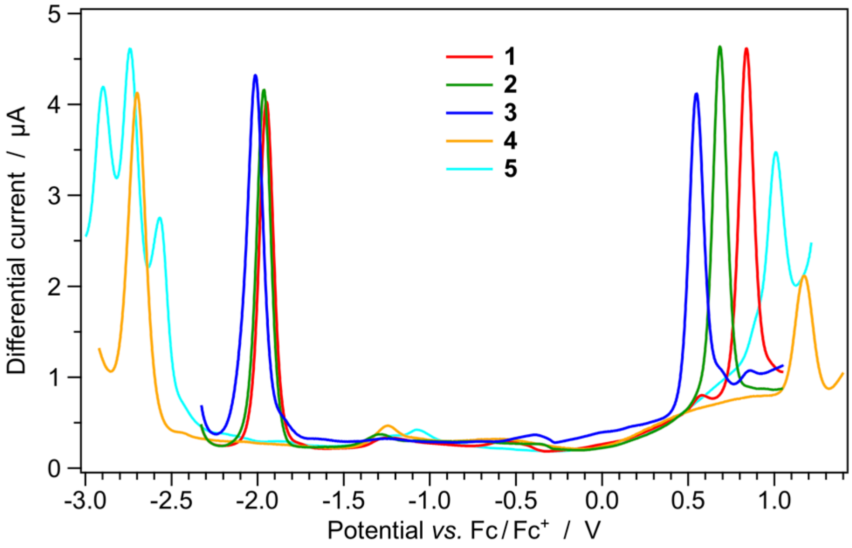

Figure S27. Square-wave voltammograms of **1**–**5** (0.5 mM) in room-temperature acetonitrile solution (with 0.1 M $TBAPF_6$ as supporting electrolyte).

Table S1. Direct comparison of the electrochemical data obtained by cyclic voltammetry and square-wave voltammetry for **1**–**5** in acetonitrile solution (0.5 mM) + 0.1 M $TBAPF_6$ at 298 K.

	Cyclic voltammetry [a]			*Square-wave voltammetry*		
	E_{ox} (ΔE_p) [b] [V (mV)]	E_{red} (ΔE_p) [b] [V (mV)]	ΔE_{redox} [c] [V]	E_{ox} [V]	E_{red} [V]	ΔE_{redox} [c] [V]
**1**	+ 0.837 (72)	– 1.948 (65)	2.785	+0.837	– 1.949	2.786
**2**	+ 0.687 (68)	– 1.964 (64)	2.651	+0.684	– 1.965	2.649
**3**	+ 0.553 (78)	– 2.014 (73)	2.567	+0.547	– 2.013	2.560
**4**	+ 1.16 (*irr.*)	– 2.70 (*irr.*)	3.86	+1.17	– 2.70	3.87
**5**	+ 1.01 (*irr.*)	– 2.57 (*irr.*), – 2.76 (*irr.*)	3.58	+1.01	– 2.57, – 2.74, – 2.90	3.58

All potentials are measured *vs.* the ferrocene/ferrocenium couple, used as internal reference.

[a] Data taken from Table 1 of the main text and herein reported again to allow a better data comparison. [b] The value in brackets is the peak-to-peak separation (ΔE_p); redox processes are reversible, unless otherwise indicated (*irr.*). [c] $\Delta E_{redox} = E_{ox} - E_{red}$.

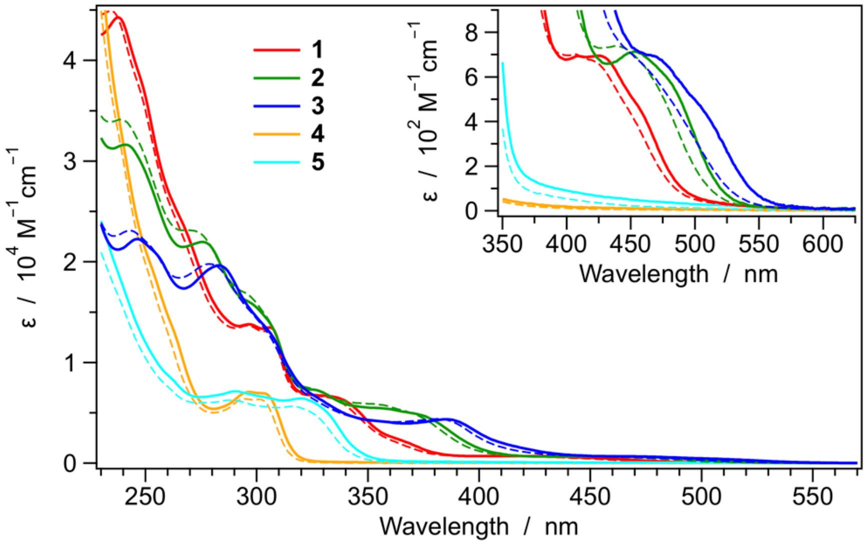

Figure S28. Absorption spectra of complexes **1**–**5** in room-temperature dichloromethane solution (solid), compared with the same spectra recorded in acetonitrile solution (dashed). Lowest-energy transitions are magnified in the inset.

Table S2. Calculated NTOs couples describing the lowest five triplet excitations for complex **1** in acetonitrile (see Experimental Section for further details). The λ value is the natural transition orbital eigenvalue associated with each NTOs couple; orbital isovalue: 0.04 $e^{-1/2}$ $bohr^{-3/2}$.

	Transition energy [eV (nm)]	NTO couple hole → electron (λ)	Nature
$S_0 \rightarrow T_1$	2.83 (439)	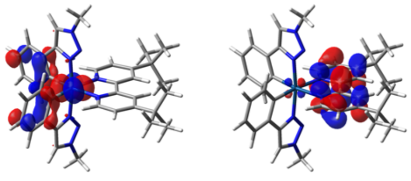<br>(99.0%)	mainly ^{3}MLCT state from the iridium ion to the ancillary ligand
$S_0 \rightarrow T_2$	2.98 (416)	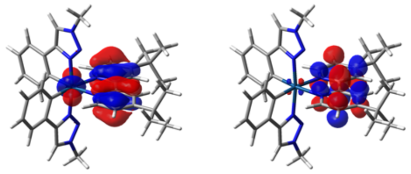<br>(82.5%)	mainly ^{3}LC on the ancillary ligand
$S_0 \rightarrow T_3$	3.13 (397)	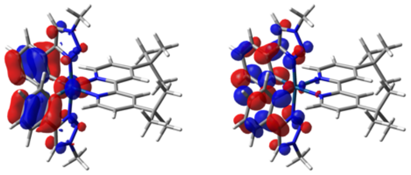<br>(55.4%)<br>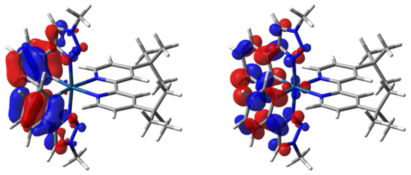<br>(29.6%)	mainly ^{3}LC on the C^N cyclometalating ligands
$S_0 \rightarrow T_4$	3.15 (394)	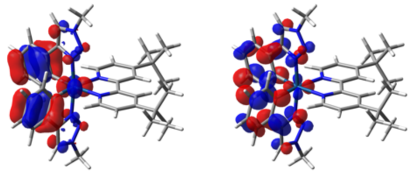<br>(46.2%)<br>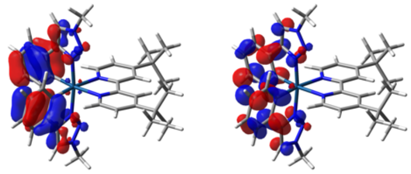<br>(37.4%)	mainly ^{3}LC on the C^N cyclometalating ligands
$S_0 \rightarrow T_5$	3.21 (386)	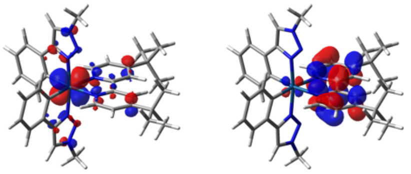<br>(94.2%)	pure ^{3}MLCT state from the iridium ion to the ancillary ligand

Table S3. Calculated NTOs couples describing the lowest five triplet excitations for **2** in acetonitrile (see Experimental Section for further details). The λ value is the natural transition orbital eigenvalue associated with each NTOs couple; orbital isovalue: 0.04 $e^{-1/2}$ $bohr^{-3/2}$.

	Transition energy [eV (nm)]	NTO couple hole → electron (λ)	Nature
$S_0 \rightarrow T_1$	2.66 (467)	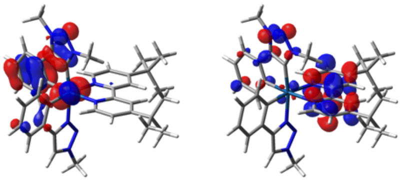 (84.1%)	mixed ^{3}MLCT/^{3}LLCT state from the iridium ion and the C^C ligand to the ancillary one
$S_0 \rightarrow T_2$	2.66 (466)	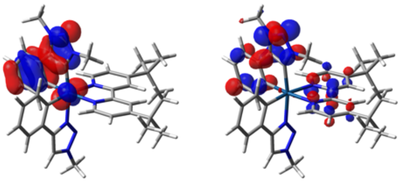 (82.1%)	mainly ^{3}LC on the C^C: cyclometalating ligand
$S_0 \rightarrow T_3$	2.91 (425)	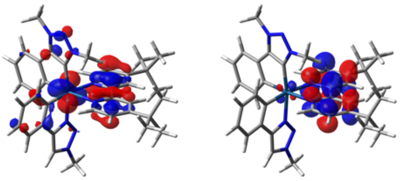 (88.1%)	mixed ^{3}MLCT/^{3}LC involving the iridium ion and the ancillary ligand
$S_0 \rightarrow T_4$	2.99 (415)	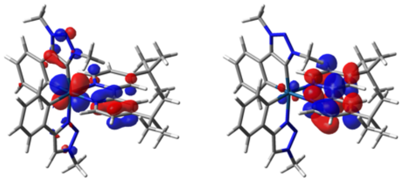 (92.3%)	mixed ^{3}MLCT/^{3}LC involving the iridium ion and the ancillary ligand
$S_0 \rightarrow T_5$	3.13 (396)	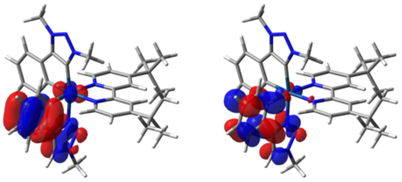 (83.9%)	mainly ^{3}LC on the C^N cyclometalating ligand

Table S4. Calculated NTOs couples describing the lowest five triplet excitations for **3** in acetonitrile (see Experimental Section for further details). The λ value is the natural transition orbital eigenvalue associated with each NTOs couple; orbital isovalue: 0.04 $e^{-1/2}$ $bohr^{-3/2}$.

	Transition energy [eV (nm)]	NTO couple hole → electron (λ)	Nature
$S_0 \rightarrow T_1$	2.57 (483)	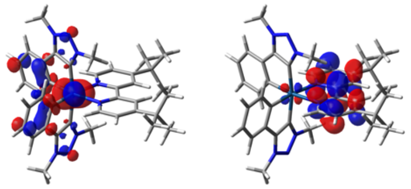<br>(98.5%)	mainly ^{3}MLCT state from the iridium ion to the ancillary ligand
$S_0 \rightarrow T_2$	2.68 (463)	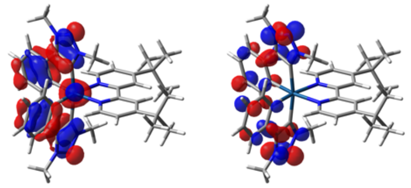<br>(55.3%)<br>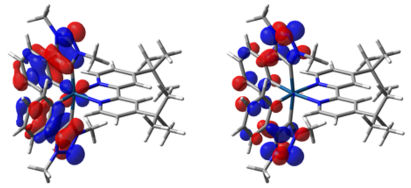<br>(39.6%)	mainly ^{3}LC on the C^C: cyclometalating ligands
$S_0 \rightarrow T_3$	2.68 (463)	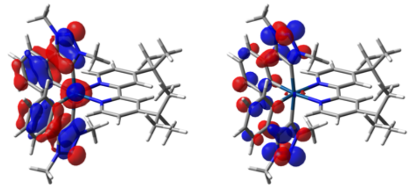<br>(48.4%)<br>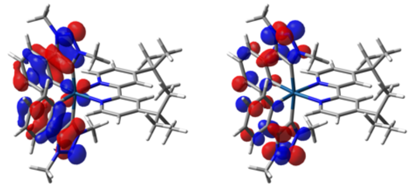<br>(46.4%)	mainly ^{3}LC on the C^C: cyclometalating ligands
$S_0 \rightarrow T_4$	2.79 (444)	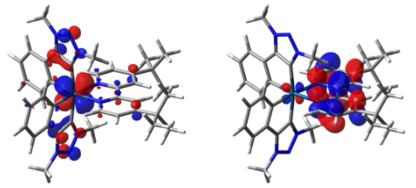<br>(97.8%)	pure ^{3}MLCT state from the iridium ion to the ancillary ligand
$S_0 \rightarrow T_5$	2.87 (432)	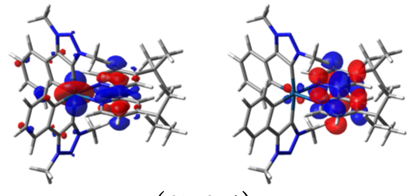<br>(87.2%)	mixed ^{3}MLCT/^{3}LC involving the iridium ion and the ancillary ligand

Table S5. Calculated NTOs couples describing the lowest four triplet excitations for **4** in acetonitrile (see Experimental Section for further details). The λ value is the natural transition orbital eigenvalue associated with each NTOs couple; orbital isovalue: 0.04 $e^{-1/2}$ $bohr^{-3/2}$.

	Transition energy [eV (nm)]	NTO couple<br>hole → electron<br>(λ)	Nature
$S_0 \rightarrow T_1$	3.20 (387)	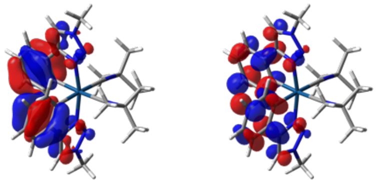<br>(42.1%)<br>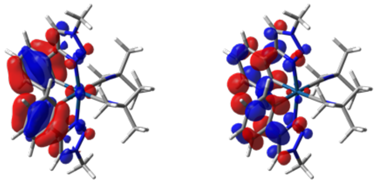<br>(38.8%)	mainly ^{3}LC on the C^N cyclometalating ligands
$S_0 \rightarrow T_2$	3.21 (386)	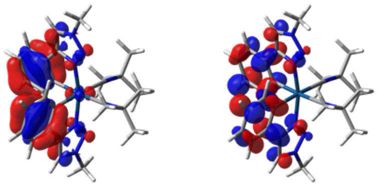<br>(43.0%)<br>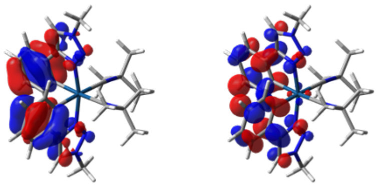<br>(38.1%)	mainly ^{3}LC on the C^N cyclometalating ligands
$S_0 \rightarrow T_3$	3.82 (324)	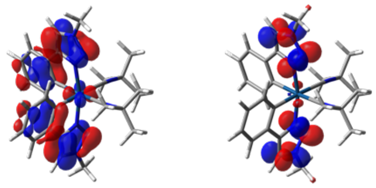<br>(51.4%)<br>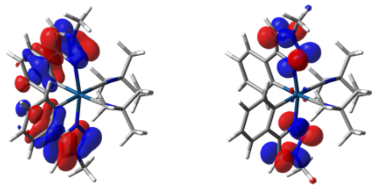<br>(45.2%)	other ^{3}LC on the C^N cyclometalating ligands
$S_0 \rightarrow T_4$	3.83 (324)	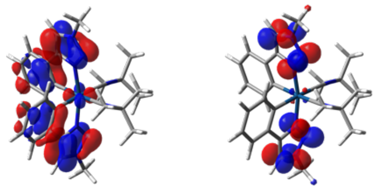<br>(53.2%)	other ^{3}LC on the C^N cyclometalating ligands

		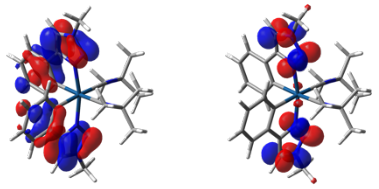 (44.7%)	

Table S6. Calculated NTOs couples describing the lowest four triplet excitations for **5** in acetonitrile (see Experimental Section for further details). The λ value is the natural transition orbital eigenvalue associated with each NTOs couple; orbital isovalue: 0.04 $e^{-1/2}$ $bohr^{-3/2}$.

	Transition energy [eV (nm)]	NTO couple hole → electron (λ)	Nature
$S_0 \rightarrow T_1$	2.80 (443)	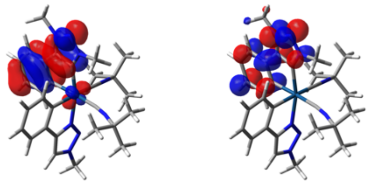 (92.5%)	mainly ^{3}LC on the C^C: cyclometalating ligand
$S_0 \rightarrow T_2$	3.21 (386)	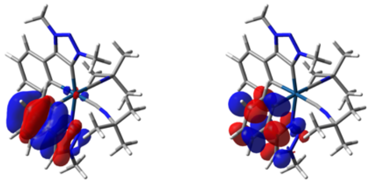 (80.6%)	mainly ^{3}LC on the C^N cyclometalating ligand
$S_0 \rightarrow T_3$	3.55 (349)	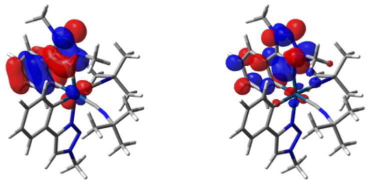 (62.7%) 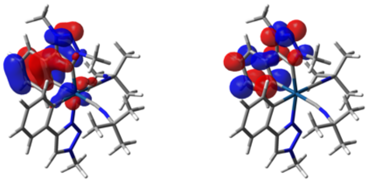 (20.1%)	other ^{3}LC on the C^C: cyclometalating ligand
$S_0 \rightarrow T_4$	3.82 (324)	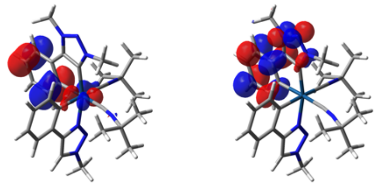 (90.4%)	other ^{3}LC on the C^C: cyclometalating ligand with internal CT character

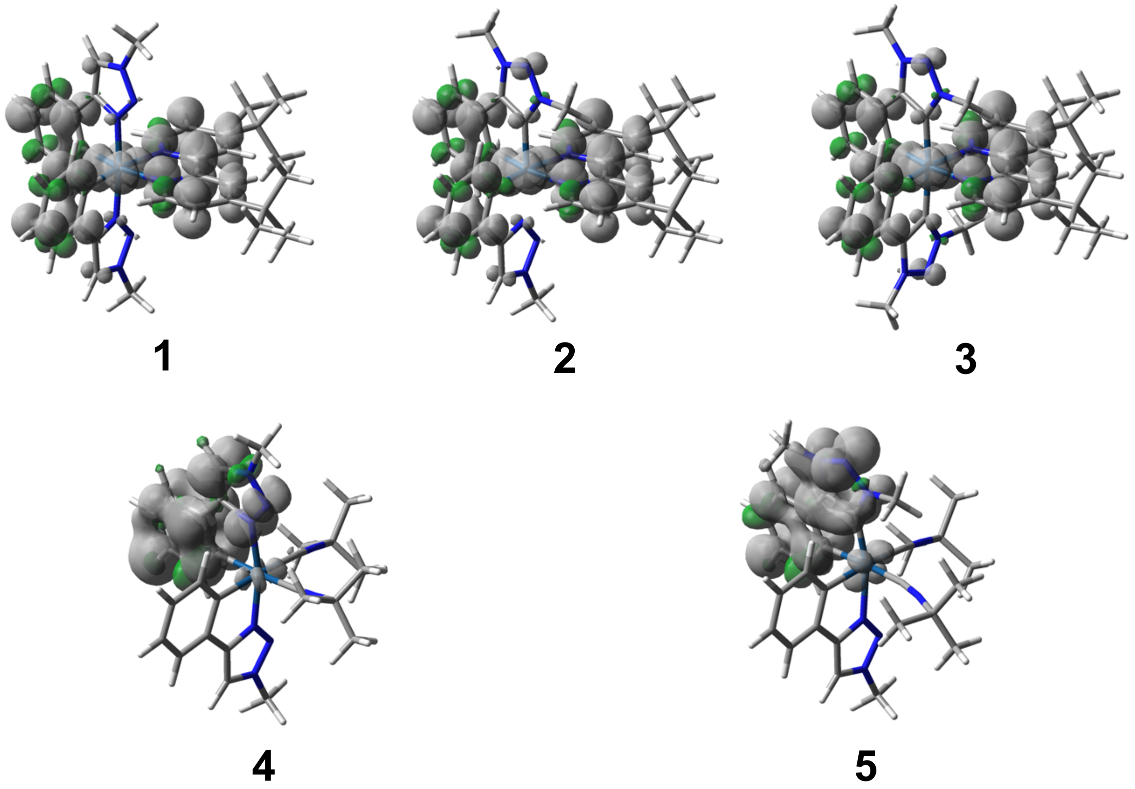

Figure S29. Spin-density distribution of the lowest triplet state (*i.e.*, T_1) of **1**–**5** in their fully-optimized geometry, computed in acetonitrile (isovalues: 0.002 e bohr^{-3}). For all the complexes equipped with the dtbbpy ancillary ligand (*i.e.*, **1**–**3**), the lowest-lying triplet state displays a ^{3}MCLT nature; for complexes **4** and **5** with *tert*-butyl isocyanide ancillary ligands, T_1 is a ^{3}LC state centered on the C^N and the C^C: cyclometalating ligands, respectively.

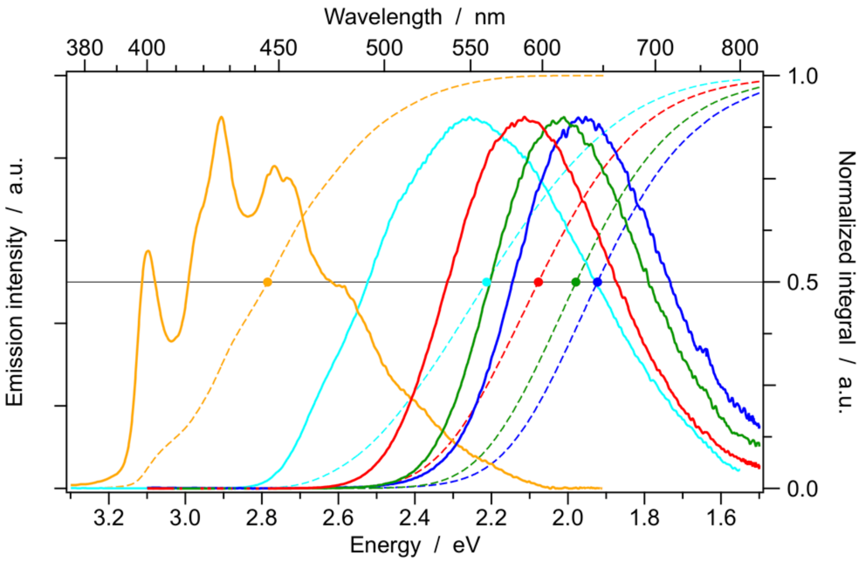

Figure S30. Corrected emission spectra of **1**–**5** in room-temperature acetonitrile solution, reported in relative quanta per energy interval. The mean-phonon energy (indicated by the dots) is calculated as the energy value at which the emission integral reaches 50% of the overall emission.

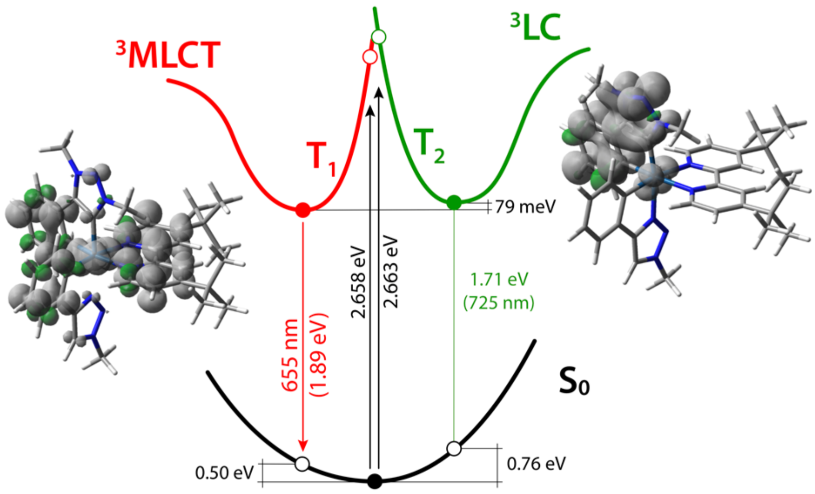

Figure S31. Schematic energy diagram reporting the ground state (S_0) and the lowest triplet states (T_1 and T_2) for **2**. Estimated absorption and emission energies are also reported, together with adiabatic energy differences between minima; the sketch is not in scale. The unpaired-electron spin-density surfaces calculated at the fully-relaxed triplet-state minima are also depicted (isovalue: 0.002 e bohr^{-3}).

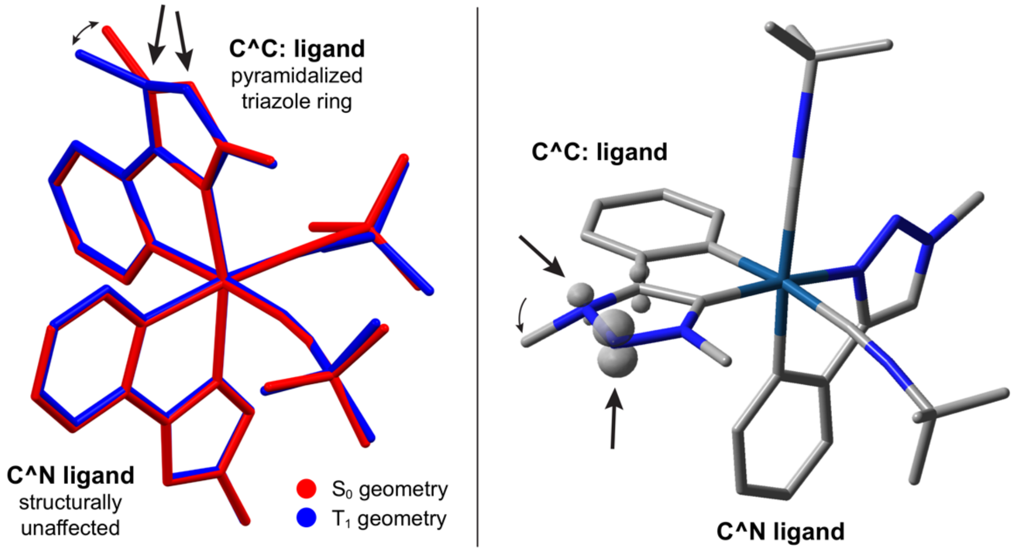

Figure S32. Left – Structural overlap (H atoms omitted for clarity) between the minimum-energy geometry of **5** in its ground state (red) and that of T_1 (blue), which is a ^{3}LC state centered on the C^C: phenyl-triazolylidene ligand (Figure S11). Right – Selected side view of **5** in its T_1 minimum geometry, showing the strong pyramidalization occurring on the N-3 atom of the triazolylidene ring in the C^C: ligand, upon relaxation. The spin-density distribution of T_1 is also reported (isovalue: 0.045 e bohr^{-3}), showing that the unpaired electron density is mainly localized on N-2 and N-3 atoms of the triazole ring.

Crystal Structure Report for compound B

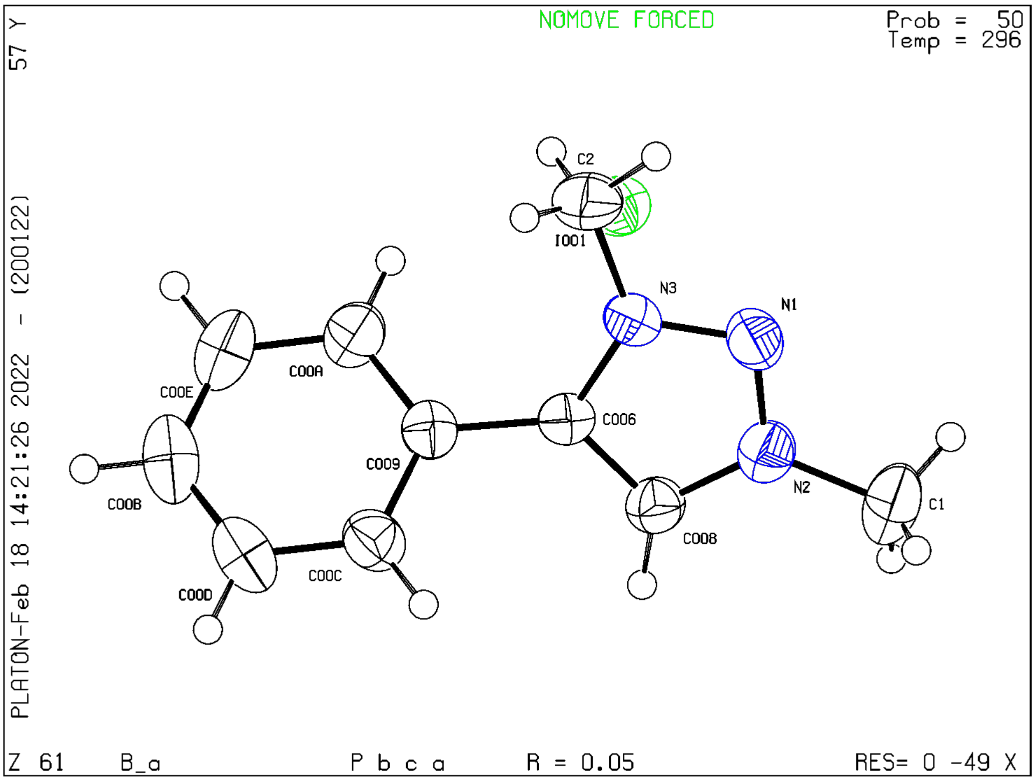

A specimen of $C_{10}H_{12}IN_3$, approximate dimensions 0.400 mm x 0.400 mm x 0.600 mm, was used for the X-ray crystallographic analysis. The X-ray intensity data were measured (λ = 0.71073 Å). The integration of the data using an orthorhombic unit cell yielded a total of 34331 reflections to a maximum θ angle of 27.50° (0.77 Å resolution), of which 2694 were independent (average redundancy 12.744, completeness = 99.6%, R_{int} = 5.49%, R_{sig} = 2.72%) and 2479 (92.02%) were greater than $2\sigma(F^2)$. The final cell constants of a = 14.1149(7) Å, b = 10.6299(5) Å, c = 15.6858(8) Å, volume = 2353.5(2) $Å^3$, are based upon the refinement of the XYZ-centroids of reflections above 20 σ(I). The calculated minimum and maximum transmission coefficients (based on crystal size) are 0.2950 and 0.4130. The structure was solved and refined using the Bruker SHELXTL Software Package, using the space group P b c a, with Z = 8 for the formula unit, $C_{10}H_{12}IN_3$. The final anisotropic full-matrix least-squares refinement on F^2 with 130variables converged at R1 = 5.03%, for the observed data and wR2 = 11.93% for all data. The goodness-of-fit was 1.245. The largest peak in the final difference electron density synthesis was 0.891 $e^-/Å^3$ and the largest hole was-2.043 $e^-/Å^3$ with an RMS deviation of 0.290 $e^-/Å^3$. On the basis of the final model, the calculated density was 1.700 g/cm^3 and F(000), 1168 e^-.

Table S7. Sample and crystal data for compound **B**.

**Identification code**	compound B	
**Chemical formula**	$C_{10}H_{12}IN_3$	
**Formula weight**	301.13 g/mol	
**Temperature**	296(2) K	
**Wavelength**	0.71073 Å	
**Crystal size**	0.400 x 0.400 x 0.600 mm	
**Crystal system**	orthorhombic	
**Space group**	P b c a	
**Unit cell dimensions**	a = 14.1149(7) Å	α = 90°
	b = 10.6299(5) Å	β = 90°
	c = 15.6858(8) Å	γ = 90°
**Volume**	2353.5(2) $Å^3$	
**Z**	8	
**Density (calculated)**	1.700 g/cm^3	
**Absorption coefficient**	2.689 mm^{-1}	
**F(000)**	1168	

Table S8. Data collection and structure refinement for compound **B**.

**Theta range for data collection**	2.73 to 27.50°	
**Index ranges**	-18<=h<=18, -13<=k<=13, -20<=l<=20	
**Reflections collected**	34331	
**Independent reflections**	2694 [R(int) = 0.0549]	
**Max. and min. transmission**	0.4130 and 0.2950	
**Structure solution technique**	direct methods	
**Structure solution program**	SHELXT 2014/5 (Sheldrick, 2014)	
**Refinement method**	Full-matrix least-squares on F^2	
**Refinement program**	SHELXL-2017/1 (Sheldrick, 2017)	
**Function minimized**	$\Sigma w(F_o^2 - F_c^2)^2$	
**Data / restraints / parameters**	2694 / 0 / 130	
**Goodness-of-fit on F^2**	1.245	
**Final R indices**	2479 data; $I>2\sigma(I)$	R1 = 0.0503, wR2 = 0.1172
	all data	R1 = 0.0519, wR2 = 0.1193
**Weighting scheme**	$w=1/[\sigma^2(F_o^2) + (0.0620P)^2 + 1.2060P]$ where $P=(F_o^2+2F_c^2)/3$	
**Extinction coefficient**	0.0680(20)	
**Largest diff. peak and hole**	0.891 and -2.043 e $Å^{-3}$	
**R.M.S. deviation from mean**	0.290 e$Å^{-3}$	

Table S9. Atomic coordinates and equivalent isotropic atomic displacement parameters (Å^2) for compound **B**. U(eq) is defined as one third of the trace of the orthogonalized U_{ij} tensor.

	x/a	y/b	z/c	U(eq)
I001	0.10824(2)	0.75309(2)	0.17589(2)	0.04431(17)
N3	0.36700(16)	0.7966(2)	0.23993(14)	0.0368(5)
C2	0.3485(3)	0.9093(3)	0.2900(2)	0.0579(8)
C1	0.4416(3)	0.6764(4)	0.0459(2)	0.0734(10)
N2	0.40998(18)	0.6966(3)	0.13352(16)	0.0451(6)
C006	0.36202(17)	0.6743(2)	0.26331(16)	0.0337(5)
N1	0.39687(17)	0.8109(3)	0.16089(17)	0.0443(6)
C008	0.39005(18)	0.6092(3)	0.19289(18)	0.0422(6)
C009	0.33569(17)	0.6265(2)	0.34777(16)	0.0371(5)
C00A	0.2568(2)	0.6704(3)	0.39123(17)	0.0491(6)
C00B	0.2887(3)	0.5280(3)	0.50606(19)	0.0616(9)
C00C	0.39059(19)	0.5321(3)	0.38407(19)	0.0473(7)
C00D	0.3661(2)	0.4825(3)	0.4629(2)	0.0573(8)
C00E	0.2342(2)	0.6209(3)	0.47074(19)	0.0602(8)

Table S10. Bond lengths (Å) for compound **B**.

N3-N1	1.318(3)	N3-C006	1.353(4)
N3-C2	1.456(4)	C2-H2A	0.96
C2-H2B	0.96	C2-H2C	0.96
C1-N2	1.461(4)	C1-H1A	0.96
C1-H1B	0.96	C1-H1C	0.96
N2-N1	1.302(5)	N2-C008	1.345(4)
C006-C008	1.362(4)	C006-C009	1.467(3)
C008-H008	0.93	C009-C00A	1.387(4)
C009-C00C	1.389(4)	C00A-C00E	1.391(4)
C00A-H00A	0.93	C00B-C00E	1.370(5)
C00B-C00D	1.372(5)	C00B-H00B	0.93
C00C-C00D	1.389(4)	C00C-H00C	0.93
C00D-H00D	0.93	C00E-H00E	0.93

Table S11. Bond angles (°) for compound **B**.

N1-N3-C006	112.5(2)	N1-N3-C2	118.1(3)
C006-N3-C2	129.4(2)	N3-C2-H2A	109.5
N3-C2-H2B	109.5	H2A-C2-H2B	109.5
N3-C2-H2C	109.5	H2A-C2-H2C	109.5
H2B-C2-H2C	109.5	N2-C1-H1A	109.5
N2-C1-H1B	109.5	H1A-C1-H1B	109.5
N2-C1-H1C	109.5	H1A-C1-H1C	109.5
H1B-C1-H1C	109.5	N1-N2-C008	112.7(2)
N1-N2-C1	119.4(3)	C008-N2-C1	127.9(3)
N3-C006-C008	104.7(2)	N3-C006-C009	126.2(2)
C008-C006-C009	129.0(3)	N2-N1-N3	104.4(3)
N2-C008-C006	105.7(3)	N2-C008-H008	127.1
C006-C008-H008	127.1	C00A-C009-C00C	119.3(3)
C00A-C009-C006	122.0(2)	C00C-C009-C006	118.6(2)
C009-C00A-C00E	119.9(3)	C009-C00A-H00A	120.1
C00E-C00A-H00A	120.1	C00E-C00B-C00D	120.1(3)
C00E-C00B-H00B	119.9	C00D-C00B-H00B	119.9
C00D-C00C-C009	120.0(3)	C00D-C00C-H00C	120.0
C009-C00C-H00C	120.0	C00B-C00D-C00C	120.2(3)
C00B-C00D-H00D	119.9	C00C-C00D-H00D	119.9
C00B-C00E-C00A	120.4(3)	C00B-C00E-H00E	119.8
C00A-C00E-H00E	119.8		

Table S12. Hydrogen atomic coordinates and isotropic atomic displacement parameters (Å^2) for compound **B**.

	**x/a**	**y/b**	**z/c**	**U(eq)**
H2A	0.3697	0.9819	0.2591	0.087
H2B	0.3818	0.9040	0.3432	0.087
H2C	0.2817	0.9162	0.3007	0.087
H1A	0.4204	0.7449	0.0108	0.11
H1B	0.4156	0.5990	0.0248	0.11
H1C	0.5095	0.6722	0.0444	0.11
H008	0.3945	0.5223	0.1871	0.051
H00A	0.2191	0.7328	0.3672	0.059
H00B	0.2734	0.4957	0.5594	0.074
H00C	0.4438	0.5023	0.3556	0.057
H00D	0.4022	0.4182	0.4866	0.069
H00E	0.1817	0.6511	0.5001	0.072

Table S13. Anisotropic atomic displacement parameters (Å^2) for compound **B**. The anisotropic atomic displacement factor exponent takes the form: $-2\pi^2[\ h^2\ a^{*2}\ U_{11} + \ldots + 2\ h\ k\ a^*\ b^*\ U_{12}\ ]$.

	**U_{11}**	**U_{22}**	**U_{33}**	**U_{23}**	**U_{13}**	**U_{12}**
I001	0.0425(2)	0.0394(2)	0.0511(2)	-0.00142(6)	0.00456(7)	-0.00374(6)
N3	0.0358(10)	0.0329(12)	0.0419(11)	0.0029(9)	-0.0012(9)	0.0012(9)
C2	0.077(2)	0.0343(14)	0.0625(19)	-0.0055(13)	0.0095(16)	0.0031(13)
C1	0.092(3)	0.082(3)	0.0457(17)	-0.0021(17)	0.0226(17)	0.002(2)
N2	0.0462(12)	0.0472(14)	0.0420(13)	-0.0002(10)	0.0061(10)	0.0001(11)
C006	0.0304(11)	0.0322(12)	0.0386(12)	-0.0020(9)	-0.0007(9)	-0.0005(9)
N1	0.0482(14)	0.0431(14)	0.0417(12)	0.0071(11)	0.0006(9)	0.0000(9)
C008	0.0449(15)	0.0371(14)	0.0445(13)	-0.0016(11)	0.0055(10)	0.0000(10)
C009	0.0375(12)	0.0368(12)	0.0370(11)	-0.0005(10)	-0.0012(9)	-0.0053(10)
C00A	0.0470(14)	0.0571(16)	0.0432(14)	-0.0031(12)	0.0025(11)	0.0031(12)
C00B	0.068(2)	0.076(2)	0.0399(14)	0.0094(14)	-0.0018(13)	-0.0230(17)
C00C	0.0476(15)	0.0424(14)	0.0519(16)	0.0066(12)	0.0012(11)	-0.0007(10)
C00D	0.0632(18)	0.0541(17)	0.0546(17)	0.0184(14)	-0.0087(14)	-0.0086(15)
C00E	0.0551(17)	0.081(2)	0.0444(15)	-0.0064(15)	0.0124(13)	-0.0067(16)
